# The critical care management of spontaneous intracranial hemorrhage: a contemporary review

**DOI:** 10.1186/s13054-016-1432-0

**Published:** 2016-09-18

**Authors:** Airton Leonardo de Oliveira Manoel, Alberto Goffi, Fernando Godinho Zampieri, David Turkel-Parrella, Abhijit Duggal, Thomas R. Marotta, R. Loch Macdonald, Simon Abrahamson

**Affiliations:** 1 Department of Medical Imaging, Interventional Neuroradiology, St. Michael’s Hospital—University of Toronto, 30 Bond Street, Toronto, ON M5B 1W8 Canada; 2 Department of Critical Care Medicine, Trauma & Neurosurgical Intensive Care Unit, St. Michael’s Hospital—University of Toronto, Toronto, ON Canada; 3 Neuroscience Research Program, Keenan Research Centre for Biomedical Science of St. Michael’s Hospital, Toronto, ON Canada; 4 Department of Medicine, Division of Respirology (Critical Care), University Health Network, Toronto, ON Canada; 5 Interdepartmental Division of Critical Care Medicine and Department of Medicine, University of Toronto, Toronto, ON Canada; 6 Intensive Care Unit, Hospital das Clínicas, Universidade de São Paulo, São Paulo, Brazil; 7 Intensive Care Unit, Hospital Alemão Oswaldo Cruz, São Paulo, Brazil; 8 Medical Intensive Care Unit, Department of Pulmonary, Allergy, and Critical Care, Respiratory Institute, Cleveland Clinic, Cleveland, OH USA; 9 Cleveland Clinic Lerner College of Medicine of Case Western Reserve University, Cleveland, OH USA; 10 Department of Surgery, Division of Neurosurgery, St. Michael’s Hospital—University of Toronto, Toronto, ON Canada; 11 Department of Anesthesiology, St. Michael’s Hospital—University of Toronto, Toronto, ON Canada

## Abstract

Spontaneous intracerebral hemorrhage (ICH), defined as nontraumatic bleeding into the brain parenchyma, is the second most common subtype of stroke, with 5.3 million cases and over 3 million deaths reported worldwide in 2010. Case fatality is extremely high (reaching approximately 60 % at 1 year post event). Only 20 % of patients who survive are independent within 6 months. Factors such as chronic hypertension, cerebral amyloid angiopathy, and anticoagulation are commonly associated with ICH. Chronic arterial hypertension represents the major risk factor for bleeding. The incidence of hypertension-related ICH is decreasing in some regions due to improvements in the treatment of chronic hypertension. Anticoagulant-related ICH (vitamin K antagonists and the newer oral anticoagulant drugs) represents an increasing cause of ICH, currently accounting for more than 15 % of all cases. Although questions regarding the optimal medical and surgical management of ICH still remain, recent clinical trials examining hemostatic therapy, blood pressure control, and hematoma evacuation have advanced our understanding of ICH management. Timely and aggressive management in the acute phase may mitigate secondary brain injury. The initial management should include: initial medical stabilization; rapid, accurate neuroimaging to establish the diagnosis and elucidate an etiology; standardized neurologic assessment to determine baseline severity; prevention of hematoma expansion (blood pressure management and reversal of coagulopathy); consideration of early surgical intervention; and prevention of secondary brain injury. This review aims to provide a clinical approach for the practicing clinician.

**Electronic supplementary material:**

The online version of this article (doi:10.1186/s13054-016-1432-0) contains supplementary material, which is available to authorized users.

## Background

Spontaneous intracerebral hemorrhage (ICH) is defined as nontraumatic bleeding into the brain parenchyma [1, 2], which can extend into the ventricles and into the subarachnoid space [3]. ICH is the second most common subtype of stroke [3], accounting for 10–50 % of all cases [4, 5], depending on the population, race, and region studied [6]. According to the “Global Burden of Diseases, Injuries, and Risk Factors” report, there were 5.3 million cases and over 3.0 million deaths secondary to ICH worldwide in 2010 [6, 7]. The case-fatality rate ranges from 35 % at 7 days to 59 % at 1 year [8–10]. Half of fatal cases occur in the first 48 hours after presentation [11, 12]. Survivors are often left with severe disability [9], with less than 40 % of patients regaining functional independence [3]. The epidemiology of ICH may change in the future with better control of risk factors such as hypertension [13], but the use of newer anticoagulation therapies may influence the acute management and possibly prognosis of the disease [14, 15].

ICH has been traditionally described as the subtype of stroke with the poorest prognosis [10]. However, recent observational reports suggested that self-fulfilling prognostic pessimism may lead to withdrawal of life support in patients who otherwise may have had an acceptable clinical outcome if managed aggressively [11]. Part of the pessimism surrounding the prognostication of hemorrhagic stroke is hypothesized to be a tendency not to consider factors such as age, previous comorbidities, etiology of the bleeding, and socioeconomic factors [12], which are known to affect outcome [16].

## Etiology and risk factors

The most important modifiable risk factor in spontaneous ICH is chronic arterial hypertension [17]. Deep perforator arteries in the pons, midbrain, thalamus, basal ganglia, and deep cerebellar nuclei, chronically damaged by hypertension, are the most common locations for hypertensive bleeding [18, 19]. Chronic hypertension is present in 50–70 % of patients who develop ICH [20]. Patients with a systolic blood pressure (SBP) ≥160 mmHg or a diastolic blood pressure ≥110 mmHg have a 5.5 (95 % CI 3.0–10.0) times increased rate of ICH, compared with normotensive patients [21]. Besides hypertension, cerebrovascular amyloid deposition (i.e., cerebral amyloid angiopathy) is associated with ICH in older patients [22]. Intracranial hemorrhage associated with cerebral amyloid angiopathy seldom occurs in subjects younger than 60 years of age. The incidence significantly increases thereafter, and is almost always associated with a lobar hemorrhage [23]. Coagulopathies (i.e., the use of antithrombotic or thrombolytic agents, congenital or acquired factor deficiencies) and systemic diseases, such as thrombocytopenia, are possible causes of ICH. The use of oral anticoagulants, especially vitamin K inhibitors (i.e., warfarin), has increased coagulopathy-associated ICH in recent years, accounting for more than 15 % of all cases [14, 24].

Psychosocial, ethnic, and economic factors play a role in the prevalence of cerebral hemorrhage, with ICH being twice as common in low-income and middle-income countries compared with high-income countries [2, 19, 25]. Other identified risk factors for ICH include age (i.e., each decade from 50 years of age is associated with a 2-fold increase in the incidence of ICH) and an elevated alcohol intake [20].

Etiologies of ICH to always consider include: intracranial aneurysms (typically presenting as subarachnoid hemorrhage); arteriovenous malformations (ICH is the first presentation of AVMs in 60 % of cases); cerebral venous sinus thrombosis and venous infarction; brain tumors (<5 % of all ICH cases) including cerebral metastasis (e.g., lung cancer, melanoma, renal cell carcinoma, thyroid carcinoma, and choriocarcinoma) and primary CNS tumors (e.g., glioblastoma multiforme and oligodendrogliomas); and drugs of abuse (e.g., cocaine, amphetamines). Because of the differing etiologies of ICH, a rapid and accurate diagnosis of the underlying etiology of ICH is essential to direct appropriate management strategies.

## Initial evaluation and clinical stabilization

**Fig. 1:**
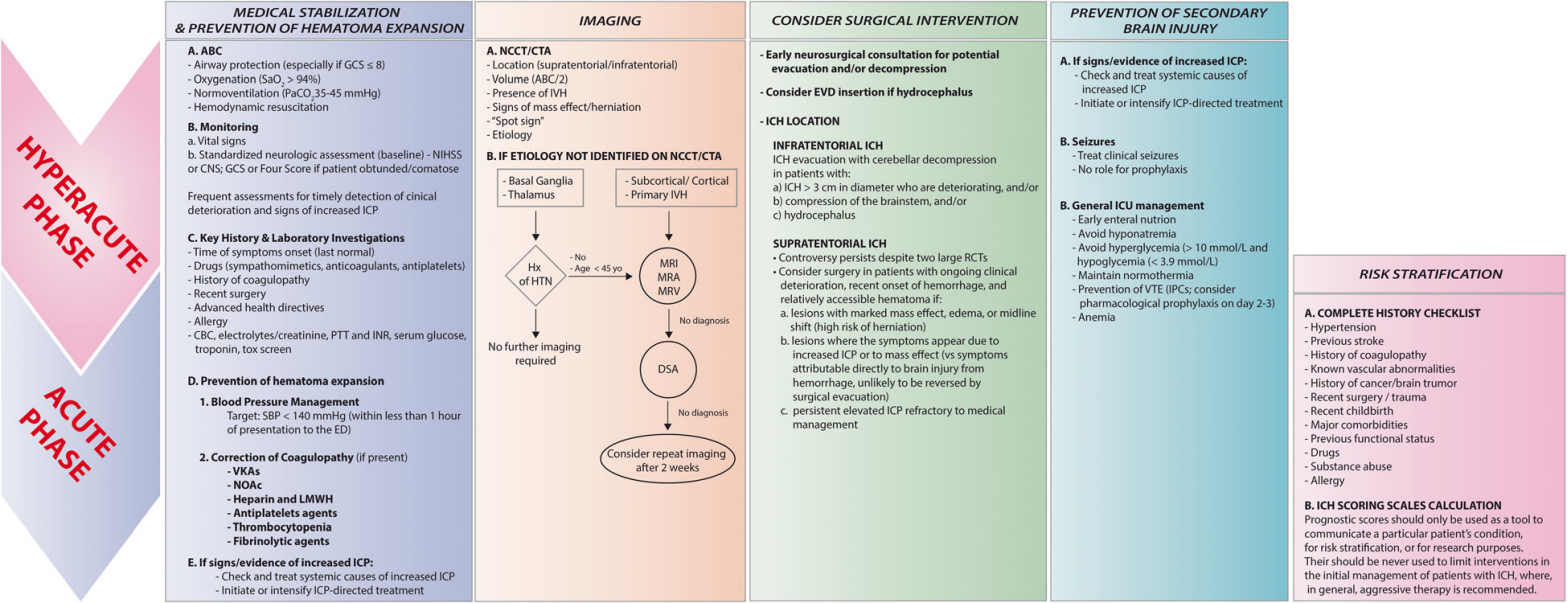
Principles of ICH management. *GCS* Glasgow Coma Scale, *SaO2* Oxygen arterial saturation, *PaCO2* partial pressure of carbon dyoxide, *ICP* intracranial pressure, *CBC* Complete Blood Count, *PTT* Partial Thromboplastin Time, *INR* international normalised ratio, *VKAs* Vitamin K inhibitors, *NOACs* newer oral anticoagulants, *LMWH* lower molecular weight heparin, *HTN* hypertension, *NCCT* non contrast computed tomography, *CTA* computed tomography angiography, *MRI* magnetic resonance imaging, *MRA* Magnetic Resonance Angiography, *MRV* Magnetic Resonance Venogram, *DSA* digital subtraction angiography, *ICH* intracerebral hemorrhage, *IVH* intraventricular hemorrhage, *NIHSS* National Institutes of Health Stroke Scale, *SBP* systolic blood pressure, *EVD* external ventricular drain

According to the AHA/ASA guidelines [9] and the Emergency Neurological Life Support protocols [26], spontaneous intracranial hemorrhage is a medical emergency and should be managed accordingly. The initial management should focus on the following principles (Fig. 1):

1. ABC’s. Initial assessment and stabilization of airway patency, breathing, and circulation.
2. Neuroimaging. Once clinical stability is achieved, an urgent imaging study for rapid and accurate diagnosis should be performed.
3. Standardized neurologic assessment to determine baseline severity. The National Institutes of Health Stroke Scale (NIHSS), if the patient is awake or drowsy, or the Glasgow Coma Scale (GCS), if the patient is obtunded or comatose, should be performed and clearly documented.
4. Blood pressure management, reversal of coagulopathy, and evaluation of the need for early surgical intervention.
5. Frequent neurological examinations, at least every hour [27, 28], to detect early clinical deterioration and signs of increased intracranial pressure (ICP) should be part of the routine initial management algorithm.

## Emergency radiologic diagnosis

The clinical presentation of ICH does not differ from acute ischemic stroke, with the sudden onset of a focal neurological deficit. However, certain clinical findings significantly increase the probability of intracranial hemorrhage, such as coma, neck stiffness, seizures accompanying the neurologic deficit, diastolic blood pressure > 110 mmHg, vomiting, and headache. Cervical bruit and prior transient ischemic attack decrease the probability of hemorrhagic stroke. However, no single clinical finding or combination of clinical findings can be considered absolutely diagnostic [29]. Neuroimaging is therefore crucial in establishing the diagnosis, and also for consideration of the underlying etiology. Current guidelines recommend either noncontrast computed tomography (CT) or magnetic resonance imaging (MRI) as the initial imaging test (Class I; Level of Evidence A) [9]. CT is usually the first-line modality given its wide availability and rapidity. Noncontrast CT is highly sensitive and specific for acute blood [9]. Magnetic resonance (gradient echo or T2 susceptibility-weighted sequences) is as sensitive as noncontrast CT in detecting acute blood, and is more sensitive in detecting previous hemorrhage. The main drawbacks of MRI use in the acute setting are cost and availability [30].

Computed tomography angiography (CTA) or contrast-enhanced CT is also commonly employed in the acute setting, combined with noncontrast CT, as a strategy to detect active contrast extravasation into the hematoma (spot sign—discussed later). Additionally, once ICH is confirmed, additional imaging (e.g., CTA, magnetic resonance angiography, or digital subtraction angiography (DSA)) is recommended to search for the underlying etiology (Additional file 1: Table S1), such as vascular malformations and brain tumors (Class IIa; Level of Evidence B) [31, 32]. If cerebral venous sinus thrombosis is suspected because of radiological findings such as unusual hematoma location, relative increased edema volume, or abnormal signal in the cerebral sinuses, CT venography or magnetic resonance venography should be performed [33].

In hypertensive patients older than 65 years with a well-circumscribed hematoma located in the basal ganglia or thalamus, the yield of such studies is low (2–3 %) and the decision not to proceed with further diagnostics tests may be reasonable [31, 32]. In young non-hypertensive patients, the following findings may warrant additional work-up: presence of isolated intraventricular hemorrhage (IVH) or subarachnoid hemorrhage, noncircular hematoma shape, disproportionately excessive edema, lobar location, identification of space-occupying lesion, enlarged vessels or calcifications along the margins of the ICH, and hyperattenuation within a dural venous sinus or cortical vein along the presumed venous drainage path. DSA remains the gold standard for identifying underlying vascular lesions (e.g., cerebral aneurysms, arteriovenous malformations), but CTA has shown an accuracy of 89–100 % for determining secondary causes of ICH [34, 35]. A recent study comparing CTA with DSA in nonhypertensive patients younger than 45 years old demonstrated excellent negative and positive predictive values of CTA and CT venography (97.3 % and 100 %, respectively) to establish or exclude vascular causes of ICH [36].

### ICH volume, IVH, and hematoma location

The volume of blood on the initial noncontrast CT image has a strong independent association with outcome. A hematoma volume of 30 ml represents a cutoff point for increased mortality [37, 38] and worse functional outcome [37]. The ICH volume can be estimated using the ABC/2 formula [39], “where A is the greatest hemorrhage diameter by CT, B is the diameter 90° to A, and C is the approximate number of CT slices with hemorrhage multiplied by the slice thickness” [40]. Additionally, when the hematoma volume is combined with the initial level of consciousness assessed by the GCS, it can accurately predict 30-day mortality [37]. Patients with an ICH volume of ≥60 ml on the initial CT image and GCS ≤ 8 have a predicted 30-day mortality >90 %, compared with a mortality of <20 % for patients with hematoma volume < 30 ml and GCS ≥ 9 [37].

Both the presence and ongoing expansion of IVH are powerful and independent predictors of functional outcomes after ICH [41]. IVH is present in approximately 45 % of patients with spontaneous ICH. IVH is associated with a lower probability of favorable outcome compared with absence of IVH (15 % vs 31 %, *p* <0.00001) [42]. An increase of more than 2 ml in IVH volume in the first 24 hours has been shown to be associated with an odds ratio (OR) for a poor outcome of 4.2 (95 % CI 1.06–16.63, *p* = 0.0405) [43].

**Fig. 2:**
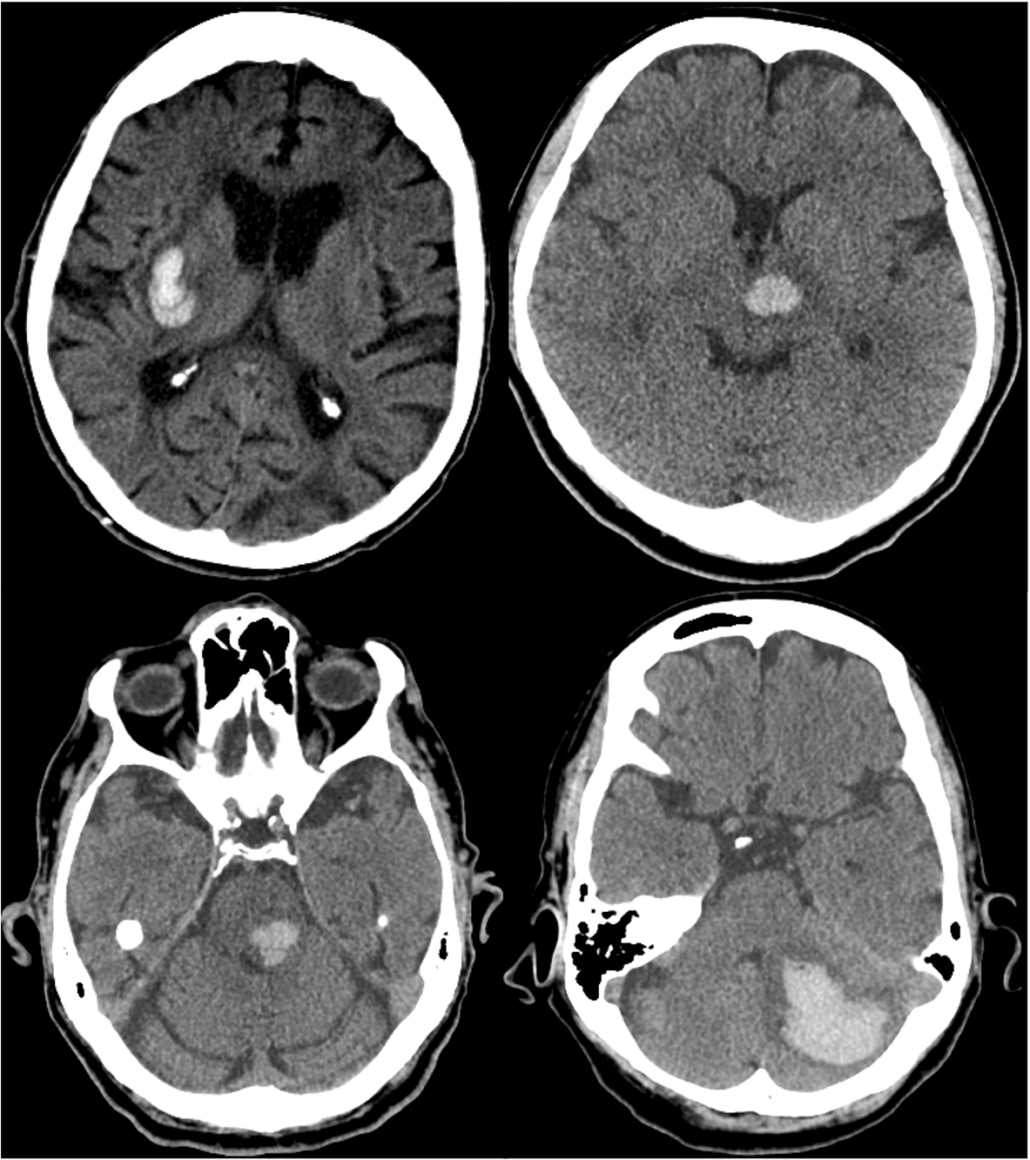
Deep intracranial hemorrhage. Common locations of hypertensive hemorrhage (*clockwise*: putamen, thalamus, cerebellum, and pons)

Hematoma location is another important factor that affects outcome and treatment [26]. The most common locations of hypertensive ICH are the basal ganglia (caudate nucleus and putamen), thalamus, deep cerebellar nuclei, midbrain, or pons (Fig. 2). Lobar hemorrhages are often associated with structural changes such as cerebral amyloid angiopathy, arteriovenous malformations, or brain tumors.

## Early neurological deterioration and hematoma expansion

ICH patients are at risk for early neurological deterioration, which is usually secondary to early hematoma expansion or the development of acute hydrocephalus secondary to IVH [44, 45]. The definition of early neurological deterioration varies across studies, but it is usually described as worsening from the initial neurological examination (e.g., a change in the initial GCS or NIHSS scores) or progression to death [46]. Early neurological deterioration occurs in up to 40 % of patients within 48 hours, and is associated with poorer long-term prognosis [46]. Mayer et al. [44] described a prospective cohort of 46 noncomatose patients (GCS ≥ 8) with ICH. Fifteen patients (33 %) developed neurological deterioration, the majority (8/15, 53 %) during the first day of hospitalization. Patients with neurological deterioration had larger hematoma volume (mean volume: 45 ml vs 16 ml, *p* < 0.01) and mass effect on initial CT (60 % vs 19 %, *p* < 0.01). The 30-day mortality was 47 % in patients with neurological deterioration compared with 3 % in those patients who did not acutely worsen (*p* = 0.001). While the two most important predictors on hospital admission of early neurological deterioration are the hematoma volume and the presence of IVH, other factors such as glucose concentration, fibrinogen levels, and elevated SBP have been described [46].

Hematoma expansion is a major determinant of early neurological deterioration, poor outcome, and death [47–49]. Brott et al. [45] found that 26 % of ICH patients developed substantial hemorrhage growth (defined as ≥33 % increase from baseline hematoma volume) between baseline and 1-hour CT scans (i.e., within 4 hours of symptom onset). Additionally 12 % of the patients developed hematoma growth between the 1-hour and 20-hour CT scans. Hemorrhage growth was significantly associated with early neurological deterioration. Hematoma expansion is an independent determinant of poor outcome and mortality [47], regardless of hematoma expansion definition [48]. The early occurrence of hematoma growth and subsequent neurological deterioration highlights the importance of frequent neurological examinations and early repeat CT scanning, which can alter medical patient care or may trigger surgical interventions [28].

### Prediction of hematoma expansion

Because hematoma expansion is a major determinant of mortality and functional outcome, it could be potentially beneficial to identify those patients at highest risk of hematoma expansion. Prediction scores have been published to predict hematoma expansion in ICH (Additional file 1: Table S5) [50–52]. Prediction scores share several common factors: shorter time from ICH onset to CT; warfarin use; and evidence of spot sign on CTA (see later). The risk of hematoma expansion varies from 3.4–7.1 % in patients with no risk factors [50–52] to 70–85.5 % in patients obtaining the maximum score [50–52].

**Fig. 3:**
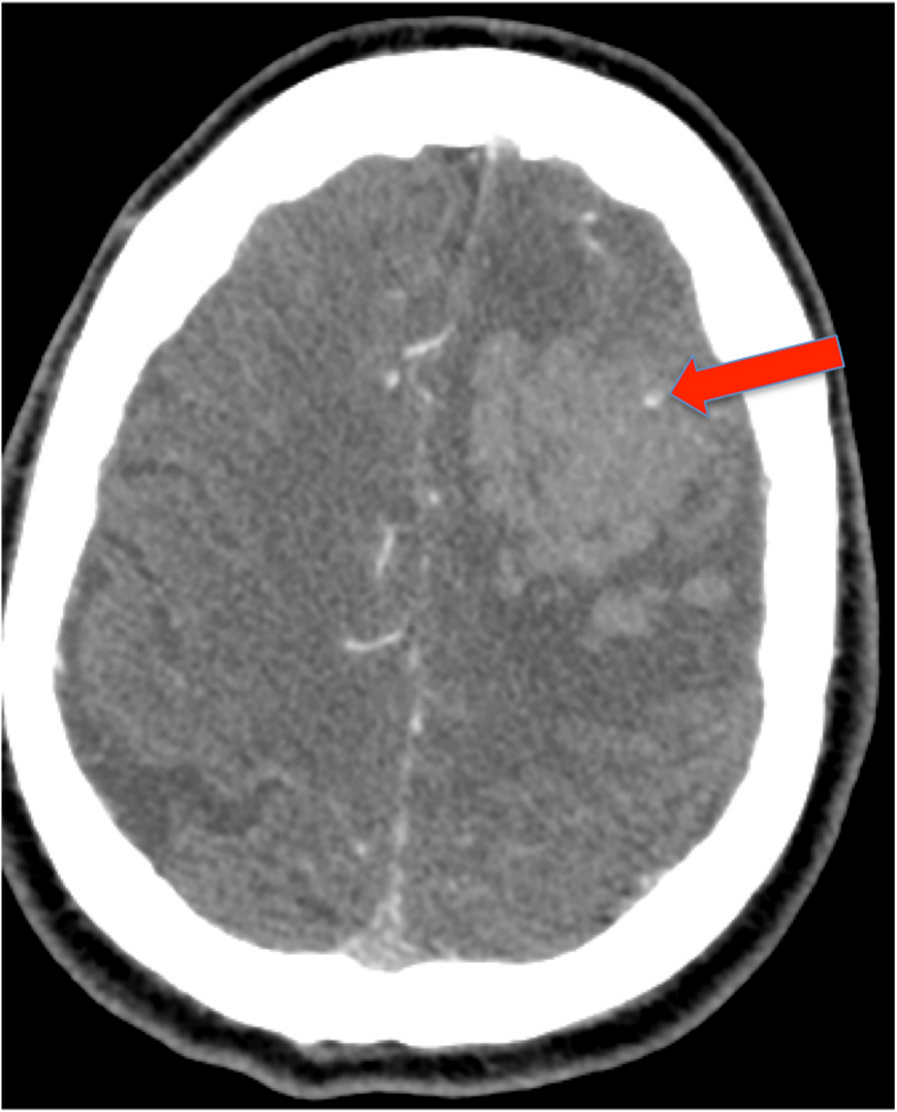
Spot sign. *Initially* described as contrast extravasation on CTA, the term has evolved to encompass foci of enhancement within the hematoma on CTA (*red arrow*)

1. Time from ICH onset to CT. As discussed previously, hematoma expansion tends to occur early in the course of ICH [45], with the majority (26 %) of significant hematoma growth happening between baseline and 1-hour CT scans, compared with only 12 % between 1-hour and 20-hour CT scans. It is not surprising that a shorter time between ICH onset and CT scan would appear to be a predictor of hematoma expansion, because CT scans performed more than 6 hours after ICH onset would probably miss hematoma growth that may have already occurred [53].
2. Patients on warfarin (or with International Normalized Ratio (INR) > 1.5) have an adjusted OR of 4.04 (95 % CI 1.85–9.13) for hematoma expansion compared with patients not on warfarin. Warfarin-related ICH is discussed in the anticoagulant-associated ICH (AAICH) section.
3. “Spot sign” (Fig. 3). Initially described as contrast extravasation on CTA [54–56], the term spot sign has evolved to encompass foci of enhancement within the hematoma on CTA [57]. Presently, the term “contrast extravasation” should only be used to describe the presence of contrast within the hematoma on post-contrast CT [58, 59]. The identification of the spot sign (Fig. 3), and its sensitivity in predicting hematoma expansion, is dependent on several technical aspects of imaging acquisition, such as the concentration of the contrast agent, the speed of scanners, and, importantly, the timing between the contrast injection and the image acquisition [60, 61]. The spot sign can be divided into the early spot sign and the delayed spot sign. The early spot sign is detected on the first-pass CTA, usually acquired in the arterial phase within 30 seconds after contrast injection. The delayed spot sign is detected on the second-pass CTA, or post-contrast CT. The second-pass CTA images (or venous phase of CTA), acquired 40 seconds to 3 minutes after contrast injection, increase the yield of identifying a spot sign if not visualized on the first-pass CTA [58, 59, 62–64]. In a systematic review and meta-analysis [53] of 14 studies of first-pass CTA, the spot sign had a sensitivity of 53 % (95 % CI 49–57 %), a specificity of 88 % (95 % CI 86–89 %), a positive likelihood ratio of 4.70 (95 % CI 3.28–6.74), and a negative likelihood ratio of 0.44 (95 % CI 0.34–0.58) for predicting hematoma expansion. When a first-pass CTA was combined with a post-contrast CT (data available for three studies) better accuracy was achieved, with a sensitivity of 92 % (95 % CI 78–98 %), a specificity of 82 % (95 % CI 74–88 %) a positive likelihood ratio of 4.89 (95 % CI 3.29–7.27), a negative likelihood ratio of 0.10 (95 % CI 0.04–0.31), a diagnostic OR of 52.62 (95 % CI 14.46–191.51), and a receiving operating curve (ROC) of 0.94 (standard error 0.05) [57, 58, 65]. Interestingly, spot sign detected in the arterial phase of CTA is associated with greater absolute hematoma expansion [64]. These results support the use of CT protocols that include non-contrast CT, followed by both CTA and post-contrast study [53]. Identification of a spot sign on CT may have several clinical implications:

  A. Identification of contrast extravasation and the spot sign are potent and independent predictors of hematoma expansion [60]. In the multicenter prospective “Prediction of hematoma growth and outcome in patients with ICH using the CT-angiography spot sign” (PREDICT) study [66], the presence of spot sign was associated with a relative risk of 2.3 (95 % CI 1.6–3.1) for hematoma expansion, defined as an absolute increase > 6 ml or a relative increase > 33 % from baseline ICH volume. However, identification of spot sign does not necessarily infer definite hematoma expansion. A spot sign score has been developed to help predict hematoma expansion [62]. The score includes: number of spot signs (1–2 or ≥3), maximum axial dimension (1–4 mm or ≥5 mm), and maximum attenuation in Hounsfield Units (120–179 HU or ≥180 HU). A score of 0 indicates that no spot sign was identified on CTA, and it has been associated with minimum risk of hematoma expansion (2 %). In patients with the maximum score (4 points), the risk for hematoma expansion approaches 100 % [62].
  B. CTA spot sign is associated with both functional outcome and mortality. In the PREDICT study [66] patients with evidence of a spot sign on CTA had a median 3-month modified Rankin Scale (mRS) of 5 (severe disability—patient bedridden, incontinent, and requiring constant nursing care and attention), in contrast to a median mRS of 3 (moderate disability—patient requiring some help, but able to walk without assistance) in spot-sign-negative patients (*p* < 0.001). The 3-month mortality was 43.4 % in the spot-sign-positive group, compared with 19.6 % in the spot-sign-negative patients (HR 2.4, 95 % CI 1.4–4.0, *p* = 0.002). Likewise, in the spot sign score study [63], the presence of spot sign was associated with an increased risk of in-hospital mortality (55.6 %, OR 4.0, 95 % CI 2.6–5.9, *p* < 0.0001) and with an unfavorable functional outcome at 3 months (50.8 %, OR 2.5, 95 % CI 1.4–4.3, *p* < 0.0014), defined as mRS ≥ 4 (moderate severe disability—patient unable to walk unassisted and unable to attend to own bodily needs without assistance).
  C. Patients with positive spot sign on CTA may benefit from more aggressive treatment to reduce hematoma expansion, which may decrease mortality and improve functional outcome [60]. Studies are enrolling spot-sign-positive patients for treatment with recombinant factor VIIa—Spot Sign for Predicting and Treating ICH Growth (STOP-IT) (ClinicalTrials.gov NCT00810888), and Spot Sign Selection of Intracerebral Hemorrhage to Guide Hemostatic Therapy (SPOTLIGHT) (ClinicalTrials.gov NCT01359202)— and for aggressive antihypertensive treatment (SCORE-IT: NIH–NINDS R01NS073344) [67] (Additional file 1: Table S4).

## Clinical severity assessment

Routine use of ICH clinical grading scales to assess baseline severity is useful in the standardization of initial assessment and in communication between providers. Scoring systems may allow for outcome risk stratification based on patients’ presentation characteristics (Class I recommendation) [9]. Several clinical grading scales have been developed to risk-stratify and predict outcome after spontaneous ICH [68]. The ICH score (Table 1) [38] is the most widely used and externally validated ICH grading scale [68]. The ICH score was derived from a retrospective analysis of 152 spontaneous ICH patients who presented to the University of California, San Francisco. Five independent predictors of 30-day mortality were identified and used to build the score: level of consciousness according to the GCS; age; ICH volume; IVH; and infra-tentorial location of ICH (Table 1) [38]. The GCS at the time of transfer from the ED to the ICU (or to the operating room) was found to be the strongest independent predictor of 30-day mortality, and consequently given the heaviest weight in the score [38]. The other four components have similar strength of association to outcome, and therefore the same weight in the grading scale. The choice of an ICH volume of 30 ml was based on previously published data [37]; larger volumes (≥60 ml) did not improve the score’s performance, possibly due to significant confounding effects of other score components (e.g., GCS) on larger hematomas [38]. The higher the score, the higher the mortality, which ranges from 0–10 % in patients with an ICH score of 0 to 100 % in patients with an ICH score ≥ 5 [68]. However, the use of clinical grading scales such as the ICH score should never be used in isolation to limit interventions in the very acute initial management of patients with ICH.

**Table 1:** Original ICH score and predicted 30-day mortality according to total score

Component	Points	Total ICH score	30-day mortality (%)
Glasgow Coma Scale			
3–4	2	0	0–10
5–12	1		
13–15	0		
Age (years)			
≥80	1	1	7–13
<80	0		
ICH volume (ml)			
≥30	1	2	30–44
<30	0		
Presence of intraventricular hemorrhage			
Yes	1	3	56–78
No	0		
Infra-tentorial origin of ICH			
Yes	1	4	70–100
No	0		
Total ICH score	0–6	5–6	100

The five independent predictors of 30-day mortality according to the original ICH score are displayed in the first column (Glasgow Coma Scale, age, ICH volume, intraventricular hemorrhage, and infra-tentorial location of ICH). The total score is the sum of the five components, varying from 0 to 6 points (column 3). The higher the total score (column 3), the higher the predicted 30-day mortality (column 4)

*ICH* intracerebral hemorrhage

## Patient disposition

Patients with ICH present management challenges from both a general medical and a neurological perspective, and they are at high risk of early deterioration [44, 45]. These patients may therefore benefit from initial monitoring and management in dedicated neuroscience ICUs or dedicated stroke units (Class I recommendation) [9, 69, 70]. In the INTERACT 2 study, fewer than 40 % of both study groups were cared for in an ICU, with the majority of patients admitted to a stroke unit rather than an ICU [71]. In a Swedish study including 105,043 patients (all types of acute stroke) from 86 hospitals, care in a stroke unit for patients with ICH was associated with lower risk of death or institutional living after 3 months (OR 0.60, 95 % CI 0.54–0.68) compared with other types of wards [72]. ICH patients were among the subgroups with the best relative effect (hazard ratio (HR) for death 0.61, 95 % CI 0.58–0.65). A systematic review and meta-analysis of eight clinical trials comparing stroke unit care with general ward care (2657 patients), showed that stroke unit care reduced death or dependency (risk ratio (RR) 0.81, 95 % CI 0.471–0.92, *p* =0.0009, *I*^2^ = 60 %) [70]. Patients requiring advanced monitoring and complex care such as external ventriculostomy drainage, ICP monitoring, mechanical ventilation, or multimodal neuromonitoring often require admission to the ICU.

## Medical management of ICH

Several issues regarding medical and surgical management of ICH remain unanswered. Recent clinical trials examining hemostatic therapy, blood pressure control, and hematoma evacuation have advanced our understanding of ICH management.

### Blood pressure management

Chronic hypertension is the main risk factor for the development of spontaneous ICH, which makes blood pressure (BP) lowering physiologically intuitive as a strategy to prevent hematoma expansion. However, physiologic concern has been that excessive blood pressure reduction could decrease cerebral perfusion pressure (CPP) in the ischemic penumbra. Clinical studies, however, have shown that this concern over the zone of perihematoma ischemia is not well substantiated clinically [73, 74]. Kate et al. [75] randomized ICH patients to two different SBP targets (aggressive group <150 mmHg, and conservative group <180 mmHg). Patients underwent CT perfusion study 2 hours after randomization, and the raw imaging data were used to calculate cerebral blood flow (CBF), maximum oxygen extraction fraction (OEF^max^), and the maximum cerebral metabolic rate of oxygen (CMRO_2_). Despite significant difference in SBP levels between the two groups (140.5 ± 18.7 mmHg vs 163.0 ± 10.6 mmHg, *p* < 0.001), perihematoma CBF, OEF^max^, and CMRO_2_ were not affected by aggressive BP lowering [75]. Recently published clinical data have further helped clarify the issue of BP thresholds in ICH. In the INTERACT pilot study [76], spontaneous ICH patients within 6 hours of onset were randomly assigned to early intensive BP-lowering strategy (target SBP = 140 mmHg) or “classic” BP management (target SBP = 180 mmHg). The study showed that acute aggressive BP lowering was feasible and safe, with a marginal attenuation in hematoma growth (1.7 ml). This pilot study was followed by the INTERACT 2 trial (a phase III trial), which enrolled 2794 patients with spontaneous ICH within 6 hours of hemorrhage [71]. Patients were randomized to the SBP targets (aggressive treatment <140 mmHg vs standard target <180 mmHg). No differences in the primary composite outcome (mortality or major disability) were found (52 % vs 55.6 %, *p* = 0.06). Moreover, early intensive BP reduction was not associated with decreased in hematoma growth, the main mechanism by which aggressive BP treatment was believed to improve outcome. However, a predefined ordinal analysis showed lower mRS scores with intensive treatment (OR for greater disability 0.87, 95 % CI 0.77–1.00, *p* = 0.04). Two possible conclusions can be drawn from this study: aggressive blood pressure lowering (SBP < 140 mmHg, ideally within 1 hour from presentation) seems to be safe in the acute phase of ICH (Class I; Level of Evidence A); and a lower blood pressure target might have an impact on long-term outcome, even if smaller than anticipated (Class IIa; Level of Evidence B) [9]. The small effect on hematoma growth and composite outcome observed in the INTERACT 2 trial may be explained by small hematoma volumes (11 ml), and, more importantly, by significant delay in BP treatment initiation (median of 4 hours from ictus) and delayed achievement in blood pressure target in the treatment group (6 hours of treatment). Therefore, it may be extrapolated that approximately 10 hours passed from hemorrhage onset to target BP achievement. By that time, hematomas would probably have already expanded. Additionally, only 34 % of patients in the treatment group achieved the goal of SBP < 140 mmHg within 1 hour. The Antihypertensive Treatment of Acute Cerebral Hemorrhage (ATACH) 2 trial has been published recently [77], and compared two targets of SBP management in the acute phase of ICH; SBP between 110 and 139 mmHg vs 140 and 179 mmHg. The trial was stopped early for futility after the prespecified interim analysis, and included 1000 patients (sample size calculated: 1280 subjects). No difference in the rate of death and disability was found, and the intervention group experienced higher rates of renal adverse events within 7 days (9.0 % vs 4.0 %, *p* = 0.002) [77]. Several differences between INTERACT 2 and ATACH 2 should be mentioned (Table 2). First, the recruitment window was 6 hours in INTERACT 2 vs 3.5 hours (which was extended to 4.5 hours throughout the study) in ATACH 2. The period of blood pressure intervention was 7 days vs 24 hours in the INTERACT 2 and ATACH 2 trials, respectively. Also, the studies started from different levels of SBP on randomization: 179 mmHg vs 200 mmHg respectively. Importantly, the mean minimum SBP achieved within 2 hours in the control group of ATACH 2 was similar to the mean SBP achieved within 6 hours in the intervention group of INTERACT 2 (141 mmHg vs 139 mmHg, respectively). In summary: the control group in the ATACH 2 trial achieved similar blood pressure levels compared with the intervention group of INTERACT 2 (141 mmHg vs 139 mmHg); the ATACH 2 trial therefore compared 140 mmHg vs 129 mmHg; and the patients included in both studies had small hematomas (approximately 10 ml), making those hematomas less likely to expand [44]. Rapid intensive blood pressure lowering might still be beneficial to patients with higher risk of hematoma expansion (e.g., larger hematomas, positive spot sign) [44, 60]. In conclusion, acute lowering of SBP to 140 mmHg is safe [9], but it does not seem to improve functional outcome, and SBP levels of 130 mmHg might be associated with increased complications. Additionally, some observational studies have demonstrated the common occurrence of small ischemic lesions on diffusion-weighted MRI after ICH. The impact of these ischemic lesions on functional outcome and their relationship with acute blood pressure lowering vary across those studies, and causality is not well established [78]. Currently, no specific anti-hypertensive agent is considered universally superior. Several agents were used in the INTERACT 2 trial [71], such as an alpha-adrenergic antagonist (urapidil, 32.5 %), calcium channel blockers (nicardipine or nimodipine, 16.2 %), combined alpha- and beta-blocker (labetalol, 14.4 %), venodilators (nitroglycerin, 14.9 %), a diuretic (furosemide, 12.4 %), and arterial vasodilators (nitroprusside, 12.1 %; hydralazine 5.9 %). The ATACH 2 trial administered nicardipine by intravenous infusion, starting at a dose of 5 mg/hour, which was increased by 2.5 mg every 15 minutes (maximum dose of 15 mg/hour), until the target SBP was achieved. Intravenous labetalol was added as second-line agent, if the SBP target was not reached, despite the maximum dose of nicardipine [77]. Nicardipine and labetalol are the most common drugs used in North America; both agents appear to be safe [79], but nicardipine might be more effective in reaching and maintaining the target BP [80]. However, local drug availability and drug approval is a practical consideration.

**Table 2:** INTERACT 2 [71] vs ATACH 2 [77] studies

	INTERACT 2 (*n* = 2794)		ATACH 2 (*n* = 1000)	
	Control	Intervention	Control	Intervention
Number of enrolled patients	1430	1399	500	500
Treatment target (SBP in mmHg)	<180	<140	140–179	110–139
Inclusion criteria	GCS > 5		ICH (volume < 60 cm^3^), GCS score ≥ 5	
Primary outcome	Death or major disability (mRS = 3–6) at 3 months		Death or disability (mRS = 4–6) at 3 months	
Recruitment window	6 hours		4.5 hours	
Medications used to lower blood pressure	Urapidil: 32.5 %		Nicardipine ± labetalol	
	Nicardipine or nimodipine: 16.2 %		Intravenous diltiazem or urapidil could be used	
	Labetalol: 14.4 %			
	Nitroglycerin: 14.9 %			
	Furosemide: 12.4 %			
	Nitroprusside: 12.1 %			
	Hydralazine: 5.9 %			
Period of blood pressure intervention	7 days		24 hours	
Time goal of blood pressure lowering	1 hour		2 hours	
Mean interval between symptom onset and randomization	3.7 hours	3.7 hours	3.0 hours	3.0 hours
Systolic blood pressure at presentation (mmHg)	179 ± 17	179 ± 17	201.1 ± 26.9	200 ± 27.1
Mean systolic blood pressure achieved (mmHg)	164 within 1 hour	150 within 1 hour	141.1 ± 14.8 (2 hours)	128.9 ± 16 (2 hours)
	153 within 6 hours	139 within 6 hours		
Primary treatment failure^a^ (%)	66		12.2	
Baseline hematoma volume (ml)	11	11	10.2	10.3
Asian (%)	68.0	67.7	57.0	55.4
Death or disability (%)—mRS = 3–6	55.6	52.0	56.1	56.2
Modified Rankin Scale (%)				
0	7.6	8.1	7.1	5.0
1	18.0	21.1	19.6	19.8
2	18.8	18.7	17.3	19.1
3	16.6	15.9	18.3	17.5
4	19.0	18.1	26.5	26.0
5	8.0	6.0	4.2	5.8
6	12.0	12.0	7.1	6.9

^a^Primary treatment failure was defined as target SBP < 140 mmHg not achieved within 1 hour of randomization in the intensive-treatment group in the INTERACT 2 trial or within 2 hours in the ATACH 2 trial

*GCS* Glasgow Coma Scale, *ICH* intracerebral hemorrhage, *mRS* modified Rankin Scale, *SBP* systolic blood pressure

### Anticoagulant-associated ICH

The use of anticoagulants has significantly increased in the last decades, leading to a 3-fold increase in the incidence of anticoagulant-related intracranial hemorrhage [14]. Patients experiencing ICH on antithrombotic agents have an increased risk for hematoma expansion and higher risk of death and poor outcome [24, 81]. In AAICH, the main goals are: withholding the culprit drug; reversing the drug effect by the administration of antidote when available; and monitoring the effectiveness of anticoagulation reversal with laboratory tests. However, the laboratory correction of coagulopathy may not be associated with the reversal of coagulopathy in vivo [40].

- *Warfarin* is responsible for 9–14 % of all cases of ICH [82], with an annual risk of warfarin-related ICH of between 0.3 and 3.7 % when the INR is between 2 and 4.5 [83] (Table 3). Patients on long-term warfarin have up to an 11-fold higher risk of ICH compared with patients not taking anticoagulants [84]. Increased age (≥70 years) [84, 85], a history of chronic hypertension [85], and the concomitant use of antiplatelet therapy (APT) [86] are risk factors for warfarin-related ICH. Bleeding risk scores (e.g., HAS-BLED) may help assess the risk of major bleeding in patients with atrial fibrillation [87]. Warfarin-related ICH is also associated with larger hematoma size [88] and higher mortality rates compared with patients without coagulopathy [81, 89]. For patients with warfarin-related ICH and elevated INR (>1.4), urgent coagulopathy reversal is warranted. Vitamin K, given as a slow intravenous infusion (5–10 mg over 30 minutes), can completely reverse the warfarin effect. However, it can take up to 24 hours to completely reverse warfarin effect, and so its use as sole therapy is associated with increased risk of hematoma expansion, and is not recommended [9]. Currently, there are two other treatment options to acutely reverse the warfarin effect: the transfusion of fresh frozen plasma (FFP) or the use of prothrombin complex concentrate (PCC). FFP contains all coagulation factors but several transfusion-related complications, such as allergic reactions, possibility of infectious disease transmission, transfusion-related acute lung injury and transfusion-related circulatory overload could occur. More importantly, the time consumed to thaw, cross-match, and transfuse the appropriate dose (10–40 ml/kg) makes the use of FFP for the acute reversal of warfarin-related ICH less appealing and less effective than the use of PCC [90]. PCC is a virally inactivated preparation of concentrated coagulation factors pooled from healthy donors. Some PCC preparations contain factors II, IX and X (three-factor PCC), while some other preparations also contain higher concentrations of factor VII (four-factor PCC). Coagulation factors can be presented in the inactivated or activated forms, depending on the formulation (Additional file 1). Dosing is usually based on factor IX administered and it is usually adjusted by weight and/or INR, although fixed doses of at least 20 UI/kg of factor IX have been reported to be effective [91]. Four-factor PCC might be more effective in reversing warfarin effect than three-factor PCC [92, 93]. A list of some four-factor PCC products available in Canada, the USA, and Europe is presented in Additional file 1. PCCs are effective for rapid reversal of anticoagulation due to warfarin use and are considered the first-choice option in some guidelines [94, 95]. Hickey et al. [96] showed in a retrospective cohort study that the median time for INR reversal was significantly shorter with four-factor PCC when compared with FFP (5.7 hours vs 11.8 hours, respectively, *p* < 0.0001). Additionally, FFP was associated with higher incidence of serious adverse events, especially heart failure (19.5 % vs 9.7 %, *p* = 0.014; relative risk 2.0, 95 % CI 1.1–3.5). In a phase IIIb study, Sarode et al. randomized 202 patients with active warfarin-related major bleeding to four-factor PCC or FFP. Rapid INR reduction, defined as the INR correction (≤1.3) at 0.5 hours after the end of infusion, occurred in 62.2 % of cases with four-factor PCC and only in 9.6 % of patients who received FFP transfusion [97].
  Although the use of recombinant activated factor VII (rFVIIa) has been described as a possible option for warfarin-related coagulopathy reversal, rFVIIa does not replace the levels of other vitamin K-dependent factors and has a short half-life. Current guidelines therefore do not recommend its use for reversal of warfarin-related ICH. Additionally, it is 15 times more expensive than FFP and at least 3.5 times more expensive than PCC, and is associated with a higher risk of INR rebound [98]. In summary, patients with warfarin-related ICH and elevated INR should have vitamin K antagonist withheld, and should receive vitamin K (10 mg IV over 30 minutes) concomitantly with PCC guided by INR level or weighted-base dosing (20 UI/kg). If PCC is not available, FFP should be administered in a dose of 10-40 ml/kg. rFVIIa is not recommended in this clinical scenario.
- *Unfractionated heparin (UFH) and low molecular weight heparins (LMWHs)*. UFH prevents fibrin formation by indirectly inhibiting factors Xa and IIa (thrombin) through the activation of antithrombin. Heparin-related ICH occurs in approximately 0.1–0.2 % of patients on continuous infusion of UFH for a non-neurological indication. For patients who develop ICH while on UFH infusion, heparin reversal is warranted. Heparin infusion should be immediately interrupted and protamine sulfate should be administered at a dose of 1 mg for every 100 units of heparin given in the previous 2–3 hours (maximum single dose of 50 mg). A repeat dose of 0.5 mg of protamine per 100 units of UFH may be given if the aPTT remains elevated [90]. Similarly to UFH, LMWHs also bind and activate antithrombin but have less effect on thrombin compared with UFH, although approximately the same effect on factor Xa. Protamine appears to only partially neutralize the anti-factor Xa activity of LMWH and therefore, in case of need for reversal, this cannot be done completely. The main goal of LMWH reversal is discontinuation of the drug, although protamine use can be attempted. For enoxaparin given within 8 hours, protamine sulfate should be administered at the dose of 1 mg per 1 mg of enoxaparin (maximum single dose of 50 mg); for enoxaparin given within 8 and 12 hours, a dose of 0.5 mg of protamine per 1 mg of enoxaparin should be administered. Beyond 12 hours of enoxaparin administration, protamine administration is not suggested. For dalteparin or nadroparin, protamine sulfate should be given at a dose of 1 mg per 100 IU of dalteparin/nadroparin administered in the past 3–5 half-lives (maximum single dose of 50 mg). Factor VIIa (90 μg/kg) can be used if protamine is contraindicated or in LMWH-related ICHs that are refractory to protamine [90].
- *Antiplatelet agents*. The literature on APT and its association with outcomes after ICH is still controversial. Several reports described the association of antiplatelet agent use with hematoma expansion and worse clinical outcome, including increased mortality rate [99–101]. However, more recent studies reported that patients on antiplatelet agents prior to ICH have similar rates of hematoma expansion compared with patients not receiving these agents, and the functional outcome may be independent of antiplatelet use [102–104]. For example, in the Cerebral Hematoma and NXY-059 Treatment (CHANT) trial, a secondary analysis of the placebo arm found no association between APT use and ICH outcomes [102]. Because it remains controversial whether APT influences hematoma expansion or functional outcome, the clinical utility of its reversal is uncertain [105]. In patients with ICH and on APT, the agent should be discontinued immediately. Some observational studies suggested potential benefit from platelet transfusion [106]. Recently, Baharoglu et al. [107] published the results of the PATCH study, the first randomized, open-label, phase 3 trial investigating the effect of platelet transfusion in patients with spontaneous ICH, who were receiving APT. The study included 190 participants from the Netherlands, the UK, and France. The study showed increased odds of a shift towards death or dependence at 3 months in the group of patients receiving platelet transfusion compared with the standard care group (adjusted common OR 2.5, 95 % CI 1.18–3.56, *p* = 0.0114). Secondly, more serious adverse events were reported in patients who received platelet transfusion (42 %), compared with 29 % in patients who received standard care alone. Interestingly, the two groups showed a difference in hematoma volume, although this was not significant (13.1 (5.4–42.4) ml in the intervention group vs 8.0 (4.4–25.8) ml in the standard of care group). In the post-hoc analysis the primary outcome remained unchanged when adjusted for ICH volume at baseline, but there was an apparent potential benefit for platelet transfusion as the hematoma volume increased; however, the very small size of the population precluded a better-powered exploratory analysis. Until the results of another similar randomized trial (ClinicalTrials.gov NCT00699621) are available, platelet transfusion should be considered not beneficial but rather potentially harmful for people taking APT and such decisions should be considered on an individual basis [9]. For example, many authors recommend platelet transfusion only for patients with aspirin-associated or ADP inhibitor associated ICH and for whom an emergency neurosurgical procedure is planned [108].
  A recent pilot study evaluated the role of desmopressin (DDAVP) to improve platelet function in patients with ICH and reduced platelet activity or on aspirin therapy [109]. Desmopressin (0.4 μg/kg IV given over 30 minutes) increased platelet activity, as measured by von Willebrand factor antigen and closure times (PFA-100 with epinephrine), 1 hour after DDAVP administration. The DDAVP effect may be short lived, however, and platelet function abnormalities may return within 3 hours [110]. Based on these limited data, a single dose of DDAVP (0.4 μg/kg IV given over 30 minutes) may be considered for patients with aspirin-associated, COX-1 inhibitor-associated, or ADP receptor inhibitor-associated ICH.
- *New oral anticoagulants*
  Factor Xa inhibitors. These oral anticoagulant agents (e.g., rivaroxaban, apixaban, and edoxaban) act as direct factor Xa inhibitors and prevent factor Xa-dependent conversion of prothrombin to thrombin. Current indications include primary stroke prevention (i.e., in nonvalvular atrial fibrillation), treatment of deep vein thrombosis and pulmonary embolism, and secondary prevention of venous thromboembolism (VTE) [108, 111]. Compared with warfarin, factor Xa inhibitors have shown a lower risk of ICH (Table 3). However, currently there is no specific antidote commercially available for this class of drug, and most information on their reversal is limited to ex-vivo and in-vivo studies on healthy volunteers and animal models of bleeding. Current recommendations suggest immediate discontinuation of the drug followed by the administration of four-factor PCC (50 U/kg) or activated PCC (50 U/kg) in the case of patients presenting within 3–5 terminal half-lives of the drug or in the presence of liver failure. In case of recent ingestion (within 2 hours), 50 g of activated charcoal is recommended [108].
  Direct thrombin inhibitor reversal. Available direct thrombin inhibitors include competitive direct thrombin inhibitor (e.g., oral dabigatran), reversible direct thrombin inhibitors (argatroban and bivalirudin, both intravenous only), and irreversible direct thrombin inhibitors (e.g., desirudin SC and lepirudin IV). Their main current indications include primary stroke prevention in patients with non-valvular AF, treatment of VTE, and management of heparin-induced thrombocytopenia [108]. Data on the incidence and outcomes of direct thrombin inhibitor-related ICH is scant. Dabigatran seems to be associated with an ICH rate of 0.2–0.3 %/year, which is lower compared with warfarin [108]. Recently, a dabigatran-specific monoclonal antibody, idarucizumab (Praxbind®), has been approved for clinical use. The use of idarucizumab in dabigatran reversal was demonstrated in an interim analysis of the Reversal Effects of Idarucizumab on Active Dabigatran Study (the RE-VERSE AD study) [112]. The report included 90 patients (one-third with ICH) with uncontrolled or life-threatening bleeding, or requiring emergency surgical procedures (<8 hours) [112]. Patients received 5 g of idarucizumab divided in two doses of 2.5 g at 15-minute intervals. Idarucizumab reversed anticoagulation in 90 % of patients within 10–30 minutes of drug infusion, as assessed by the dilute thrombin time (TT) and ecarin clotting time (ECT). Therefore, for patients with dabigatran-related ICH, emergency treatment should include discontinuation of the drug, followed by two doses of 2.5 g idarucizumab IV at 15-minute intervals, if the last dose of dabigatran was ingested within 3–5 half-lives or if renal failure is present. In the occurrence of dabigatran intoxication or renal failure, the use of hemodialysis can be considered. If idarucizumab is not available, or if the ICH is related to other direct thrombin inhibitors, emergency treatment should include discontinuation of the drug followed by the administration of activated PCC (50 U/kg) or a four-factor PCC (50 U/kg), if the last dose of drug was ingested within 3–5 terminal half-lives. If the drug was taken more than 3–5 half-lives before presentation, reversal in not indicated. In the case of recent ingestion (within 2 hours), 50 g of activated charcoal is recommended. The use of rFVIIa or FFP in direct thrombin inhibitor-related intracranial hemorrhage is not recommended.
- *rFVIIa in patients without coagulopathy*. The use of rFVIIa in noncoagulopathic patients has been studied in multiple randomized trials and has been shown to reduce hematoma growth but not to improve patient survival or functional outcome. Additionally, its use is associated with an increased rate of arterial thromboembolic adverse events [113]. Currently, two clinical trials—the SPOTLIGHT trial (ClinicalTrials.gov NCT01359202) and the STOP-IT trial (ClinicalTrial.gov NCT0081088)—are underway to clarify the role of rFVIIa in ICH patients with a positive-spot sign. Current guidelines do not recommend the use of rFVIIa in unselected noncoagulopathic ICH patients [9].
- Other anticoagulant agent reversal strategies are summarized in Table 3.
  Table 4 summarizes recent studies examining hemostatic therapy, blood pressure management, and the surgical approach to ICH.

**Table 3:** Anticoagulants and reversal strategies

Drug	Target	Elimination and half-life (hours)	Rate of ICH	Monitoring coagulation tests	Antidote and reversal	Possible intervention	Guidelines
Vitamin K antagonist							
Warfarin	Factors II, VII, IX, X; proteins C, S	Hepatic metabolism	0.3–1.1 % [90]	Good linear correlation PT/INR	Vitamin K	Not dialyzable	Withhold VKA + intravenous vitamin K + replace vitamin K–dependent factors (three- or four-factor PCC IV or FFP if PCCs are not available), and correct the INR (keep INR < 1.4) (Class I; Level of Evidence C) [9]
		92 % renal elimination			Vitamin K 10 mg IV associated with 4-FPCC 20 IU/kg (or FFP = 10–15 ml/kg, if PCC is not available)		PCCs might be considered over FFP (Class IIb; Level of Evidence B) [9]
		20–60			Goal: INR < 1.4 [90]		rFVIIa is not recommended for VKA reversal in ICH (Class III; Level of Evidence C) [9]
Unfractionated heparin, LMWHs, and heparinoids							
UFH	Binds and activates antithrombin (which blocks coagulation factors Xa and IIa). By inactivating thrombin, heparin prevents fibrin formation	Renal	0.1 to 0.2 % [90, 151]	Good linear correlation aPTT	Protamine sulfate 1 mg of protamine per 100 units of UFH infused over the preceding 3 hours	Not dialyzable	Protamine sulfate—1 mg for every 100 units of heparin given in the previous 2–3 hours with a maximum single dose of 50 mg (Strong recommendation, moderate quality evidence) [90]
		0.5–2.5 (dose dependent)					If aPTT is still elevated, repeat administration of protamine at a dose of 0.5 mg protamine per 100 units of UFH (Conditional recommendation, low quality of evidence) [90]
							Reversal of prophylactic SC heparin only if aPTT is significantly prolonged (Good Practice statement) [90]
Enoxaparin	LMWH Binds and activates antithrombin (which blocks coagulation factors Xa and IIa)	40 % renal	0.2–0.5 % [98] Enoxaparin 1 mg/kg BID; bridging warfarin with target INR 2–3	Anti-factor Xa	Protamine sulfate partially reverses (60 %) LMWH effect. One mg protamine for every 1 mg enoxaparin	Not dialyzable	Strong recommendation, moderate quality evidence [90]
		4.5 hours					Protamine sulfate 1 mg per 1 mg of enoxaparin (maximum single dose of 50 mg—if enoxaparin was given within 8 hours)
							Protamine sulfate 0.5 mg of protamine per 1 mg of enoxaparin (if enoxaparin was given within 8–12 hours)
							After 12 hours, protamine is not needed
Dalteparin	LMWH Binds and activates antithrombin (which blocks coagulation factors Xa and IIa)	Renal	Not established	Anti-factor Xa	Protamine sulfate partially reverses (60 %) LMWH effect. One mg protamine for every 100 anti-Xa IU dalteparin	Not dialyzable	Protamine sulfate 1 mg per 100 IU of dalteparin administered in the past 3–5 half-lives (maximum 50 mg) (Strong recommendation, moderate quality evidence) [90]
		2.5 hours					
		3.7–7.7 hours with RF					
Nadroparin	LMWH Binds and activates antithrombin (which blocks coagulation factors Xa and IIa)	Renal	Not established	Anti-factor Xa	Protamine sulfate partially reverses (60 %) LMWH effect. One mg protamine for every 100 anti-Xa IU nadroparin	Not dialyzable	Protamine sulfate 1 mg per 100 IU of nadroparin administered in the past 3–5 half-lives (maximum 50 mg) (Strong recommendation, moderate quality evidence) [90]
		3.5 hours					
Pentasaccharides							
Fondaparinux (Aristra®)	Binds with antithrombin and potentiates inhibition of free factor Xa, preventing formation of the prothrombinase complex	50–77 % renal	Not established	Anti-factor Xa	None	Activated PCC (FEIBA 20 units/kg)	aPCC 20 IU/kg (Conditional recommendation, low-quality evidence) [90]
		17–21 hours				Dialyzable (clearance increased by 20 %)	rFVIIa (90 μg/kg) if aPCC is not available (Conditional recommendation, low-quality evidence) [90]
		Prolonged in older patients and in RF					Protamine sulfate is not recommended (Strong recommendation, low-quality evidence) [90]
Direct thrombin (factor IIa) inhibitors							
Argatroban (Acova®)	Competitive direct inhibition of thrombin (factor IIa) including thrombin-mediated platelet activation and aggregation	No renal excretion 0.75 hours (prolonged in hepatic dysfunction)	Not established	aPTT, ACT	None	Activated PCC (FEIBA 50–80 units/kg) Antifibrinolytic agent (e.g., tranexamic acid, epsilon-aminocaproic acid) Hemodialysis (approximately 20 % over 4 hours)	aPCC (50 units/kg) or four-factor PCC (50 units/kg) (Conditional recommendation, low-quality evidence) [90] rFVIIa or FFP are not recommended in direct thrombin inhibitor-related ICH (Strong recommendation, low-quality evidence) [90]
Bivalirudin	Reversible direct inhibition of thrombin (factor IIa) including thrombin-mediated platelet activation and aggregation	20 % renal	0.1 % [151]	ECT (PT, aPTT, ACT has nonlinear prolongation)	None	Activated PCC (FEIBA 50–80 units/kg)	
		0.5 (prolonged in renal impairment)				Antifibrinolytic agent (e.g., tranexamic acid, epsilon-aminocaproic acid)	aPCC (50 units/kg) or four-factor PCC (50 units/kg) (Conditional recommendation, low-quality evidence)
		GFR 30–59, 34 minutes				Hemodialysis (approximately 25 % over 4 hours)	rFVIIa or FFP are not recommended in direct thrombin inhibitor-related ICH (Strong recommendation, low-quality evidence)
		GFR 10–29, 57 minutes					
Dabigatran (Pradaxa®)	Reversible direct inhibition of thrombin (factor IIa) including thrombin-mediated platelet activation and aggregation	>80 % renal	0.30 % (150 mg) [98]	Modified TT/ECT/prolongs PT linearly with increasing serum levels, while aPTT is affected in a nonlinear way	Idarucizumab or Praxbind® (humanized antibody fragment against dabigatran), in two doses of 2.5 g IV 15 minutes apart	Activated PCC (FEIBA 50–80 units/kg)	Idarucizumab 5 g IV in two divided doses if dabigatran was administered within 3–5 half-lives and no RF (Strong recommendation, moderate quality of evidence) or in the presence of RF leading to continued drug exposure beyond the normal 3–5 half-lives (Strong recommendation, moderate quality of evidence)
		12–17 hours	0.23 % (110mg) [98]			Activated charcoal if last dose was taken < 2 hours Hemodialysis (approximately 57 % over 4 hours) Antifibrinolytic agent (e.g., tranexamic acid, epsilon-aminocaproic acid)	Hemodialysis if idarucizumab is not available (Conditional recommendation, low-quality data)
		16.6 hours in mild RF	ICH distribution: 46 % intraparenchymal, 45 % SDH, and 8 % SAH [90]				
		18.7 hours in moderate RF					
		27.5 hours in severe RF					
		34.1 hours in patients on hemodialysis					
Desirudin	Irreversible direct inhibition of thrombin (factor IIa) including thrombin-mediated platelet activation and aggregation	40–50 % renal 2 hours (12 hours with renal impairment)	Not established	aPTT	None	Dialyzable	aPCC (50 units/kg) or four-factor PCC (50 units/kg) (Conditional recommendation, low-quality evidence) [90] rFVIIa or FFP are not recommended in direct thrombin inhibitor-related ICH (Strong recommendation, low-quality evidence) [90]
Direct factor Xa inhibitors							
Apixaban (Eliquis®)	Prevents factor Xa-mediated conversion of prothrombin to thrombin	Mainly fecal 27 % renal 8–14 hours	Apixaban 5 mg twice daily 0.33 %/year [98]	Anti-factor Xa There are scant data regarding the effect of apixaban on traditional coagulation tests	Currently, there is no FDA-approved specific antidote for this class of anticoagulants Antidotes under investigation: – Aripazine (PER977—synthetic small molecule) – Andexanet (PRT064445—recombinant modified factor Xa protein)	Unactivated four-factor PCC (50 units/kg). If not available, a three-factor PCC can be used. Activated charcoal if last dose was taken < 2 hours Antifibrinolytic agent (e.g., tranexamic acid, epsilon-aminocaproic acid) Minimal removal with dialysis (decreased by 14 % over 4 hours)	Activated charcoal (50 g) within 2 hours of ingestion (Conditional recommendation, very low-quality evidence) [90] aPCC (50 units/kg) or Four-factor PCC (50 U/kg) or activated PCC (50 U/kg) if ICH happened within 3–5 half-lives of drug or if liver failure (Conditional recommendation, low-quality evidence) [90] Four-factor PCC or activated PCC over rFVIIa (Conditional recommendation, low-quality evidence) [90]
Rivaroxaban (Xarelto®)	Prevents factor Xa-mediated conversion of prothrombin to thrombin	66 % renal 28 % fecal 7 to 11 hours	Rivaroxaban 20 mg daily - < 0.5 % /year	Anti-factor Xa Rivaroxaban levels linearly increase PT and aPTT levels	Currently, there is no FDA-approved specific antidote for this class of anticoagulants Antidotes under investigation: – Aripazine (PER977—synthetic small molecule) – Andexanet (PRT064445—recombinant modified factor Xa protein)	Unactivated four-factor PCC (50 units/kg). If not available, a three-factor PCC can be used. Activated charcoal if last dose was taken < 2 hours Antifibrinolytic agent (e.g., tranexamic acid, epsilon-aminocaproic acid) Not dialyzable (rivaroxaban is highly protein bound)	Activated charcoal (50 g) within 2 hours of ingestion (Conditional recommendation, very low-quality evidence) [90] Four-factor PCC (50 U/kg) or activated PCC (50 U/kg) if ICH happened within 3–5 half-lives of drug or if liver failure (Conditional recommendation, low-quality evidence) [90] Four-factor PCC or activated PCC over rFVIIa (Conditional recommendation, low-quality evidence) [90]
Edoxaban	Prevents factor Xa-mediated conversion of prothrombin to thrombin	50 % renal 10–14 hours	Edoxaban 60 mg daily compared with warfarin (HR 0.54, 95 % CI 0.38–0.77) [90]	There are scant data regarding the effect of edoxaban on traditional coagulation tests	Currently, there is no FDA-approved specific antidote for this class of anticoagulants Antidotes under investigation: – Aripazine (PER977—synthetic small molecule) – Andexanet (PRT064445—recombinant modified factor Xa protein)	Unactivated four-factor PCC (50 units/kg). If not available, a three-factor PCC can be used. Activated charcoal if last dose was taken < 2 hours Antifibrinolytic agent (e.g., tranexamic acid, epsilon-aminocaproic acid) Not dialyzable (edoxaban is highly protein bound)	Activated charcoal (50 g) within 2 hours of ingestion (Conditional recommendation, very low-quality evidence) [90] Four-factor PCC (50 U/kg) or activated PCC (50 U/kg) if ICH happened within 3–5 half-lives of drug or if liver failure (Conditional recommendation, low-quality evidence) [90] Four-factor PCC or activated PCC over rFVIIa (Conditional recommendation, low-quality evidence) [90]
Antiplatelets							
Aspirin	Irreversible COX-1 and 2 enzyme inhibitor (inhibits thromboxane A2)	5.6–35.6 % renal 0.3 hours	It is unclear if antiplatelet therapy increases the incidence of ICH	Light Transmission Platelet Aggregation with or without Secretion	None The usefulness of platelet transfusions in ICH patients with a history of antiplatelet use is uncertain	Dialyzable DDAVP 0.4 μg/kg	DDAVP 0.4 μg/kg IV (Conditional recommendation, low-quality evidence) [90] Platelet transfusion is not recommended (Conditional recommendation, low-quality evidence) [90] Platelet transfusion for patients who will undergo a neurosurgical procedure (Conditional recommendation, moderate quality of evidence) [90] DDAVP can be used in addition to platelet transfusion in patients who will undergo neurosurgical procedure (Conditional recommendation, low-quality evidence) [90]
Clopidogrel	Irreversible inhibition of P2Y12 ADP receptor	50 % renal 46 % fecal 6–8 hours	It is unclear if anti platelet therapy increases the incidence of ICH	Light Transmission Platelet Aggregation with or without Secretion	None The usefulness of platelet transfusions in ICH patients with a history of antiplatelet use is uncertain	Not dialyzable DDAVP 0.4 μg/kg	DDAVP 0.4 μg/kg IV (Conditional recommendation, low-quality evidence) [90] Platelet transfusion is not recommended (Conditional recommendation, low-quality evidence) [90] Platelet transfusion for patients who will undergo a neurosurgical procedure (Conditional recommendation, moderate quality of evidence) [90] DDAVP can be used in addition to platelet transfusion in patients who will undergo neurosurgical procedure (Conditional recommendation, low-quality evidence) [90]
Prasugrel	Irreversible inhibition of P2Y12 ADP receptor	68 % renal 27 % fecal 2–15 hours	It is unclear if anti platelet therapy increases the incidence of ICH	Light Transmission Platelet Aggregation with or without Secretion	None The usefulness of platelet transfusions in ICH patients with a history of antiplatelet use is uncertain	Not dialyzable DDAVP 0.4 μg/kg	DDAVP 0.4 μg/kg IV (Conditional recommendation, low-quality evidence) [90] Platelet transfusion is not recommended (Conditional recommendation, low-quality evidence) [90] Platelet transfusion for patients who will undergo a neurosurgical procedure (Conditional recommendation, moderate quality of evidence) [90] DDAVP can be used in addition to platelet transfusion in patients who will undergo neurosurgical procedure (Conditional recommendation, low-quality evidence) [90]
Ticlopidine	Irreversible inhibition of P2Y12 ADP receptor	60 % renal 23 % fecal 12 hours (increases with RF to 4–5 days after repeated doses)	It is unclear if anti platelet therapy increases the incidence of ICH	Light Transmission Platelet Aggregation with or without Secretion	None The usefulness of platelet transfusions in ICH patients with a history of antiplatelet use is uncertain	Not dialyzable DDAVP 0.4 μg/kg	DDAVP 0.4 μg/kg IV (Conditional recommendation, low-quality evidence) [90] Platelet transfusion is not recommended (Conditional recommendation, low-quality evidence) [90] Platelet transfusion for patients who will undergo a neurosurgical. procedure (Conditional recommendation, moderate quality of evidence) [90] DDAVP can be used in addition to platelet transfusion in patients who will undergo neurosurgical procedure (Conditional recommendation, low-quality evidence) [90]
Dipyridamole	Reversible adenosine reuptake inhibitor	Fecal 10 hours	It is unclear if anti platelet therapy increases the incidence of ICH	Light Transmission Platelet Aggregation with or without Secretion	None The usefulness of platelet transfusions in ICH patients with a history of antiplatelet use is uncertain	Not dialyzable DDAVP 0.4 μg/kg	DDAVP 0.4 mcg/kg IV (Conditional recommendation, low-quality evidence) [90] Platelet transfusion is not recommended (Conditional recommendation, low-quality evidence) [90] Platelet transfusion for patients who will undergo a neurosurgical procedure (Conditional recommendation, moderate quality of evidence) [90] DDAVP can be used in addition to platelet transfusion in patients who will undergo neurosurgical procedure (Conditional recommendation, low-quality evidence) [90]
Ticagrelor	Reversible inhibition of P2Y12 ADP receptor	26 % renal 58 % fecal 7 hours (metabolite = 9 hours)	It is unclear if anti platelet therapy increases the incidence of ICH	Light Transmission Platelet Aggregation with or without Secretion	None The usefulness of platelet transfusions in ICH patients with a history of antiplatelet use is uncertain	Not dialyzable DDAVP 0.4 μg/kg	DDAVP 0.4 mcg/kg IV (Conditional recommendation, low-quality evidence) [90] Platelet transfusion is not recommended (Conditional recommendation, low-quality evidence) [90] Platelet transfusion for patients who will undergo a neurosurgical procedure (Conditional recommendation, moderate quality of evidence) [90] DDAVP can be used in addition to platelet transfusion in patients who will undergo neurosurgical procedure (Conditional recommendation, low-quality evidence) [90]
Cilostazol	Reversible phosphodiesterase III inhibitor, increases cAMP, inhibits ADP-induced platelet aggregation, and causes vasodilation	74 % renal 20 % fecal 10 hours	It is unclear if anti platelet therapy increases the incidence of ICH	Light Transmission Platelet Aggregation with or without Secretion	None The usefulness of platelet transfusions in ICH patients with a history of antiplatelet use is uncertain	Not dialyzable DDAVP 0.4 μg/kg The usefulness of platelet transfusions in ICH patients with a history of antiplatelet use is uncertain	DDAVP 0.4 μg/kg IV (Conditional recommendation, low-quality evidence) [90] Platelet transfusion is not recommended (Conditional recommendation, low-quality evidence) [90] Platelet transfusion for patients who will undergo a neurosurgical procedure (Conditional recommendation, moderate quality of evidence) [90] DDAVP can be used in addition to platelet transfusion in patients who will undergo neurosurgical procedure (Conditional recommendation, low-quality evidence) [90]

*aPTT* activated partial thromboplastin time, *ECT* ecarin clotting time, *ICH* intracerebral hemorrhage, *INR* International Normalized Ratio, *PCC* prothrombin complex concentrate, *PT* prothrombin time, *TT* thrombin time, *LMWH* low-molecular-weight heparin, *UFH* unfractionated heparin, *RF* renal failure, *GFR* glomerular filtration rate, *BID* two times a day, *SDH* subdural hematoma, *SAH* subarachnoid hemorrhage, *HR* heartrate, *ACT* activated clotting time, *FFP* Fresh frozen plasma, *IV* intravenous, *VKA* vitamin K antagonist, *rFVIIa* recombinant activated factor VII, *SC* subcutaneous, *DDAVP* Desmopressin

**Table 4:** Evidence-based summary

Study	Design	Intervention	Results
Blood pressure management			
Rapid Blood Pressure Reduction in Acute Intracerebral Hemorrhage [152]	Prospective randomized, single-center study *Primary outcome*: clinical deterioration (NIHSS drop ≥ 2 points) within the first 48 hours. Hematoma enlargement at 24 hours was a secondary endpoint *Eligible patients*: acute spontaneous supra-tentorial ICH within 8 hours of symptom onset *Exclusion criteria:* history of head trauma; coma with signs of herniation; coagulopathy; MAP < 110 mmHg at presentation; secondary ICH; surgical hematoma evacuation	*Blood pressure targets:* standard treatment (MAP = 110–130 mmHg) vs aggressive BP treatment (MAP < 110 mmHg) *Maximum interval from symptom onset to treatment:* 8 hours *Duration of treatment:* 48 hours *Drugs used:* – Initially, intermittent labetalol infusions (10–20 mg) – If target blood pressure was not achieved, a continuous infusion of nicardipine (5–15 mg/hour) was started – More severe cases were treated with intravenous nicardipine from the onset (i.e., initial dose, 5 mg/hour followed by titration and increases of 2.5 mg/hour every 5–15 minutes, and no bolus) – Most severe cases of hypertension were treated with sodium nitroprusside infusion at 0.3 μg/kg/minute IV infusion and titrated every few minutes to desired effect	21 patients in each group Treatment was started on average 3.2 ± 2.2 hours after symptom onset Target blood pressure was achieved within 87.1 ± 59.6 minutes in the standard group and 163.5 ± 163.8 minutes in the aggressive BP treatment group No significant differences in early neurological deterioration, hematoma and edema growth, and clinical outcome at 90 days (mRS)
INTERACT trial [76]	Open-label, multicenter, blinded outcome, randomized trial; 44 hospital sites in Australia, China, and South Korea *Primary outcome*: proportional change in hematoma volume at 24 hours *Eligible patients*: spontaneous ICH and elevated BP (≥2 measurements of 150–220 mmHg, recorded ≥ 2 minutes apart) *Exclusion criteria:* SBP > 220 mmHg or hypertensive encephalopathy; severe cerebral artery stenosis or renal failure; secondary ICH or the use of a thrombolytic agent; ischemic stroke within 30 days; GCS 3–5; significant prestroke disability or medical illness; or early planned decompressive neurosurgical intervention	*Blood pressure targets:* early intensive BP-lowering strategy (target SBP = 140 mmHg within 1 hour) vs standard approach (target SBP =180 mmHg) *Maximum interval from symptom onset to treatment:* 6 hours *Duration of treatment:* 7 days *Drugs used:* treatment conducted with locally available intravenous or oral BP-lowering agent, each included (treatment group): furosemide (35 %), urapidil (47 %), phentolamine (16 %), glycerol trinitrate (10 %), labetalol (6 %), nicardipine (5 %), hydralazine (3 %), metoprolol (1 %), nitrate patch (3 %). It also included some oral agents	203 patients randomized to intensive BP management vs 201 to standard guidelines-based management Trend toward lower hematoma growth at 24 hours in the intensive treatment group (difference 22.6 %, 95 % CI 0.6–44.5 %, *p* = 0.04; absolute difference in volume 1.7 ml, 95 % CI 0.5–3.9 ml, *p* = 0.13) No excess of neurological deterioration, other clinical outcomes, or adverse events
ATACH I trial [154]	Phase I, dose-escalation, multicenter prospective study *Eligible patients*: spontaneous ICH with admission SBP ≥ 170 mmHg on two repeat measurements at least 5 minutes apart. Symptom onset < 6 hours at the time to evaluation and initiation of treatment with IV nicardipine *Primary outcomes*: (1) Feasibility end point: SBP reduction and maintenance in the respective target range achieved (treatment success) or not (failure); (2) Safety end points: (a) neurologic deterioration (defined by a decline in the GCS score 2 or increase in NIHSS score 4 points not explained by use of sedatives or hypnotics) within 24 hours from treatment initiation; (b) serious adverse events within 72 hours	*Blood pressure targets:* – Tier 1: SBP ≥ 170 and < 200 mmHg – Tier 2: SBP ≥ 140 and < 170 mmHg – Tier 3: SBP ≥ 110 and < 140 mmHg *Maximum interval from symptom onset to treatment:* 6 hours *Duration of treatment:* 18–24 hours *Drugs used:* intravenous nicardipine infusion, initiated at 5 mg/hour, and then increased by 2.5 mg every 15 minutes as needed, up to a maximum of 15 mg/hour. Once the target SBP was achieved, the infusion rate was decreased by 1–3 mg/hour. If the SBP dropped below the specified levels, infusion was reduced by 2.5 mg/hour every 15 minutes until the drug was stopped	60 patients enrolled (Tier 1 = 18; Tier 2 = 20; Tier 3 = 22) Nine patients in Tier 3 had treatment failure Seven patients had neurologic deterioration (one, two, and four in Tiers 1, 2, and 3, respectively) One subject in Tier 2 and three in Tier 3 had serious adverse events; however, the safety-stopping rule was not activated in any of the tiers These results confirmed the feasibility and safety of early rapid lowering of BP in ICH and formed the based for the larger randomized ATACH II trial
ICH ADAPT study [74]	Multicenter, prospective, randomized, open-label, with blinded evaluation study. A block randomization design (six patients/block), stratified by onset to treatment time (≤6 and 6–24 hours) *Eligible patients*: spontaneous ICH diagnosed <24 hours after onset and SBP >150 mmHg *Exclusion criteria:* Secondary ICH (e.g., vascular malformation), planned surgical resection, or contraindications to CT perfusion (CTP; e.g., contrast allergy or renal impairment) *Primary end-point*: difference in perihematoma relative cerebral blood flow (CBF) between treatment groups as assessed by CT perfusion imaging 2 hours post randomization	*Blood pressure targets:* SBP target <150 mmHg vs <180 mmHg to be achieved within 1 hour of randomization *Drugs used:* labetalol test dose: 10 mg bolus over 1 minute. If SBP > target (150 or 180 mmHg) and heart rate (HR) > 55 bpm, the bolus was repeated (10 mg bolus in 5 minutes). 10–20 mg IV push every 5 minutes until SBP < target or HR < 55. Labetalol maximum 300 mg/24 hours Hydralazine was used only if SBP persistently > target, or if HR was below 55 Enalapril was used in the SBP < 150 mmHg target group. It was considered in patients with labile BP, who required repeated doses of labetalol and/or hydralazine. 1.25 mg IV every 6 hours PRN	75 patients enrolled Focal decreases in CBF and cerebral blood volume within the perihematoma region evident in all patients. After adjustment for baseline intraparenchymal hematoma volume and time to randomization, perihematoma relative CBF not significantly lower in patients randomized to SBP < 150 mmHg (*p* = 0.18; absolute difference 0.03, 95 % CI –0.018 to 0.078)
INTERACT2 trial [71]	International, multicenter, prospective, randomized, open-treatment, blinded end-point trial *Eligible patients*: spontaneous ICH and elevated BP (at least two SBP measurements of ≥150 and ≤220 mmHg, recorded 2 or more minutes apart) *Exclusion criteria:* structural cerebral cause for the intracerebral hemorrhage; GCS 3–5; massive hematoma with a poor prognosis; or early planned surgery to evacuate the hematoma *Primary outcome*: death or major disability (mRS3–6 at 90 days)	*Blood pressure targets:* intensive treatment (SBP <140 mmHg within 1 hour) vs guideline-recommended treatment (SBP <180 mmHg) *Maximum interval from symptom onset to treatment:* 6 hours *Duration of treatment:* 7 days *Drugs used:* treatment conducted with locally available intravenous BP-lowering agent, each included (treatment group): alpha-adrenergic antagonist (i.e., urapidil, 32.5 %), calcium-channel blocker (nicardipine or nimodipine, 16.2 %), combined alpha- and beta-blocker (labetalol, 14.4 %), nitroglycerin (14.9 %), furosemide (12.4 %), nitroprusside (12.1 %), hydralazine (5.9 %)	2839 patients enrolled at 144 hospitals in 21 countries (*n* = 1403 early intensive treatment; *n* = 1436 guideline-recommended treatment) No statistically significant difference between two groups in the rates of death or severe disability (52 % vs 55.6 %; OR with intensive treatment 0.87, 95 % CI 0.75–1.01, *p* = 0.06) A pre-specified ordinal analysis of the mRS score showed significantly lower scores with intensive treatment (OR for greater disability 0.87, 95 % CI 0.77–1.00, *p* = 0.04)
ATACH 2 [77]	International, multicenter, randomized, open label trial *Eligible patients*: ICH (volume, <60 cm^3^) and GCS ≥ 5 *Exclusion criteria:* structural cerebral cause for the intracerebral hemorrhage; GCS 3–4; massive hematoma with a poor prognosis; or early planned surgery to evacuate the hematoma *Primary outcome*: death or major disability (mRS 4–6 at 3 months)	*Blood pressure targets:* intensive treatment (SBP 110–139 mmHg within 2 hours) vs SBP between 140 and 179 mmHg) *Maximum interval from symptoms onset to treatment:* 4.5 hours *Duration of treatment:* 24 hours *Drugs used:* nicardipine IV, started at a dose of 5 mg per hour, and increased by 2.5 mg per hour every 15 minutes (maximum dose of 15 mg per hour). Intravenous labetalol was added as second-line agent, if the systolic blood pressure target was not reached	1000 patients enrolled at 110 sites in six countries (*n* = 500 intensive treatment; *n* = 500 standard treatment) No statistically significant difference in the rates of death or disability (38.7 vs 37.7 %; intensive vs standard treatment, respectively) Relative risk, 1.04; 95 % CI 0.85–1.27; analysis was adjusted for age, initial GCS score, and presence or absence of intraventricular hemorrhage The rate of renal adverse events within 7 days was significantly higher in the intensive treatment group (9.0 % vs 4.0 %, *p* = 0.002)
Intensive blood pressure reduction in acute intracerebral hemorrhage: a meta-analysis [155]	Systematic review and meta-analysis according to PRISMA guidelines. Included available randomized controlled trials that randomized patients with acute ICH to either intensive or guideline BP-reduction protocols at the time of publication	Included four studies: – Rapid Blood Pressure Reduction in Acute Intracerebral Hemorrhage [152] – INTERACT [76] – ICH ADAPT [74] – INTERACT2 [71]	3315 patients Death rates were similar between the groups (OR 5 1.01, 95 % CI 0.83–1.23, *p* ≤ 0.914) Intensive BP-lowering treatment tended to be associated with lower 3-month death or dependency (mRS grades 3–6) compared with guideline treatment (OR 5 0.87, 95 % CI 0.76–1.01, *p* = 0.062) No evidence of heterogeneity between estimates (*I* ^2^ ≤ 0 %, *p* ≤ 0.723), or publication bias in the funnel plots (*p* ≤ 0.993, Egger statistical test), was detected Intensive BP reduction was also associated with a greater attenuation of absolute hematoma growth at 24 hours (standardized mean difference ± SE: –0.110 ± 0.053, *p* = 0.038)
Hemostasis			
FAST trial [153]	Multicenter, randomized, double blinded, placebo-controlled trial *Eligible patients*: spontaneous ICH within 3 hours of symptom onset *Exclusion criteria:* GCS ≤ 5; secondary ICH; known use of anticoagulant therapy, thrombocytopenia, or coagulopathy; acute sepsis; crush injury; disseminated intravascular coagulation; pregnancy; previous disability; known recent thromboembolic disease *Primary outcome*: death or severe disability (mRS 5–6 at 90 days)	Patients randomized to single intravenous dose of rFVIIa (20 or 80 μg/kg) or placebo within 4 hours from stroke	841 patients (*n* = 268, placebo; *n* = 276, rFVII 20 μg/kg; *n* = 297, rFVII 80 μg/kg) 80 μg/kg of rFVIIa associated with significant reduction in ICH expansion (mean estimated increase in volume of ICH: 26 % placebo; 18 % 20 μg/kg; 11 % 80 μg/kg) Despite the reduction in bleeding, no significant difference in the proportion of patients with poor outcome (24 % placebo; 26 % 20 μg/kg; 29 % 80 μg/kg) More arterial thromboembolic events in the group receiving rFVII 80 μg/kg versus placebo (9 % vs 4 %, *p* = 0.04)
A meta-analysis of the efficacy and safety of recombinant activated factor VII for patients with acute intracerebral hemorrhage without hemophilia [131]	Systematic review and meta-analysis	Included five studies (one included traumatic ICH patients): – Safety and feasibility of recombinant factor VIIa for acute intracerebral hemorrhage – Recombinant activated factor VII for acute intracerebral hemorrhage – Recombinant activated factor VII for acute intracerebral hemorrhage: US phase IIA trial – Recombinant factor VIIA in traumatic intracerebral hemorrhage: results of a dose-escalation clinical trial – Efficacy and safety of recombinant activated factor VII for acute intracerebral hemorrhage	rFVIIa reduced the change in ICH volume There was no significant difference in mortality, mRS score or extended Glasgow Outcome Scale (GOS-E) score in patients treated with rFVIIa or placebo There was a significant increase in arterial thromboembolic adverse events in patients treated with rFVIIa There was an increase in deep vein thrombosis in patients with spontaneous ICH and traumatic ICH
PATCH trial [107]	Multicenter, randomized, open-label, masked end-point, parallel-group, phase 3 trial; 60 hospitals (36 Netherlands, 13 UK, 11 France) *Eligible patients*: nontraumatic supra-tentorial ICH; GCS 8–15; antiplatelet therapy for at least 7 days preceding ICH; pre-ICH mRS 0–1 *Exclusion criteria*: epidural or subdural hematoma; underlying aneurysm or arteriovenous malformation; planned surgical evacuation of ICH within 24 hours of admission; IVH more than sedimentation in the posterior horns of the lateral ventricles; previous adverse reaction to platelet transfusion; vitamin k antagonist use or knows coagulopathy; thrombocytopenia (<100,000 cells/ml) *Primary outcome*: difference in functional outcome at 3 months after randomization scored with mRS	Platelet transfusion (leukocyte-depleted, either buffycoat derived or collected by apheresis) to be initiated within 6 hours of symptom onset and 90 minutes of brain imaging – COX inhibitor, with or without adenosine-reuptake inhibitor: 1 platelet concentrate (equivalent to 5 donor units) – ADP receptor inhibitor, with or without another antiplatelet drug: 2 platelet concentrates	190 participants at 41 different sites enrolled between February 2009 and October 2015 (97 patients assigned to receive standard care with platelet transfusion and 93 assigned to standard care without transfusion) 4 patients assigned to platelet transfusion did not receive it; 2 participants assigned to standard care alone received platelet transfusion Primary outcome: higher odds of a shift towards death or dependence at 3 months in the platelet transfusion group (adjusted common OR 2.05, 95 % CI 1.18–3.56, *p* = 0.0114) Secondary analysis: more patients in the platelet transfusion group with mRS 4–6 than those in standard care group In-hospital mortality: 24 (25 %) patients assigned to platelet transfusion; 15 (16 %) patients assigned to standard care alone Pre-specified subgroup analyses (type of anti-platelet therapy; country; hematoma volume): no significant interaction Serious adverse events: 40 (42 %) patients in platelet transfusion group; 28 (29 %) patients in standard care group
Surgical treatment			
STICH [114]	International, multicenter, prospective, randomized trial *Eligible patients*: spontaneous supra-tentorial ICH within 72 hours; uncertainty about the benefits of either treatment according to responsible neurosurgeon *Exclusion criteria:* secondary ICH; cerebellar hemorrhage or extension of a supra-tentorial hemorrhage into the brainstem; severe pre-existing physical or mental disability or severe comorbidities; surgery not undertaken within 24 hours of randomization *Primary outcome:* death or disability using the extended Glasgow Outcome Scale 6 months after ictus	Patients randomized to early surgery (hematoma evacuated within 24 hours of randomization by the method of choice of the responsible neurosurgeon, combined with the best medical treatment) or to initial conservative management (best medical treatment; later surgical evacuation allowed in case of neurological deterioration)	1033 patients from 83 centers in 27 countries (*n* = 503 early surgery; *n* = 530 initial conservative treatment) Of the 468 patients randomized to early surgery analyzed at 6 months, 122 (26 %) had a favorable outcome compared with 118 (24 %) of 496 patients randomized to initial conservative treatment (OR 0.89, 95 % CI 0.66–1.19, *p* = 0.414; absolute benefit 2.3 %; relative benefit 10 %) 26 % of patients initially randomized to conservative treatment underwent surgery after an initial period of observation Subgroup analysis of patients with lobar ICH within 1 cm of the cortical surface who underwent surgery had a statistically significant increase in good outcomes compared with similar subjects in the medical arm (8 % absolute increase, *p* = 0.02)
STICH II [115]	International, multicenter, prospective, randomized, parallel group, pragmatic trial *Eligible patients*: spontaneous lobar ICH, ≤1 cm from the cortical surface of the brain, volume between 10 and 100 ml; within 48 hours of onset of ictus; best GCS motor score ≥ 5 and best GCS eye score ≥ 2 *Exclusion criteria:* secondary ICH; involvement of basal ganglia, thalamic, cerebellar, or brainstem regions; presence of IVH; severe pre-existing physical or mental disability or severe comorbidities *Primary outcome*: prognosis-based favorable or unfavorable outcome dichotomized from the Extended Glasgow Outcome Scale at 6 months after randomization	Patients randomized to early surgery (evacuation of hematoma within 12 hours of randomization) or initial conservative treatment (delayed evacuation permitted if judged clinically appropriate)	601 patients from 78 centers in 27 countries (*n* = 307 early surgery; *n* = 294 initial conservative treatment) Median time to craniotomy: 26 hours after stroke onset No difference in the primary outcome (absolute difference 3.7 %, 95 % CI –4.3 to 11.6 %; OR 0.86, 95 % CI 0.62–1.20, *p* = 0.37) In the subgroup of patients with a poor expected prognosis at enrollment (lower GCS, greater age, and larger ICH volume), early surgical intervention was associated with more favorable outcome (OR 0.49, 95 % CI 0.26–0.92, *p* = 0.02) No advantage for surgery in the good prognosis group (OR 1.2, 95 % CI 0.75–1.68, *p* = 0.57) Among patients in the initial conservative treatment group, 21 % had surgery

*BP* blood pressure, *CT* computed tomography, *GCS* Glasgow Coma Scale, *ICH* intracerebral hemorrhage, *mRS* modified Rankin scale, *NIHSS* National Institutes of Health Stroke Scale, *OR* odds ratio, *rFVIIa* recombinant activated factor VII, *SBP* systolic blood pressure, *MAP* mean arterial pressure, *IV* intravenous, *PRN* when necessary

## Surgical treatment of spontaneous intracranial hemorrhage

### Supra-tentorial hemorrhage

The benefits of clot removal have been addressed in two randomized trials. The Surgical Trial in Intracerebral Hemorrhage (STICH) randomized 1033 patients with supra-tentorial hemorrhage (lobar or ganglionic hematoma) to early surgery (within 96 hours of ictus) versus standard of care (i.e., medical management with delayed surgery if necessary) [114]. No difference in favorable functional outcome at 6 months was found (*p* = 0.414). However, the subgroup of patients with superficial ICHs (lobar hemorrhage within 1 cm of the cortical surface) who underwent surgery had better outcomes. This result prompted a second trial, STICH II, aiming at randomizing patients with superficial lobar hematomas (10–100 ml) to early surgery versus medical management with delayed surgery if necessary [115]. Patients with IVH or coma were excluded. STICH II found no difference in mortality or severe disability with early surgery (*p* = 0.37). Of note, patients with predicted poor prognosis at enrollment (estimated according to a prognostic model taking into account GCS, age, and ICH volumes: 10 × GCS − age − 0.64 × volume) were more likely to have a favorable outcome with early surgery than with initial conservative treatment (OR 0.49, *p* = 0.02). Such a benefit with early surgery was not detected in the group of patients with predicted good prognosis at enrollment (OR 1.12, *p* = 0.57) [115].

### Posterior fossa hemorrhage

Hemorrhage involving the posterior fossa (cerebellum or brainstem; Fig. 2) can be associated with life-threatening complications, such as acute hydrocephalus secondary to fourth-ventricle compression and direct brainstem compression and/or herniation through the foramen magnum. Treatment strategies include posterior fossa (suboccipital) decompressive craniectomy, external ventricular drain (EVD) insertion or conservative management. There is no randomized trial addressing the best approach or timing to manage infra-tentorial hemorrhage and the evidence available is based on class III studies. Different protocols and algorithms have been published, directing management strategies on the basis of GCS and hematoma size [116], degree of fourth-ventricle compression [117], or GCS and presence of hydrocephalus [118]. Patients with a GCS score of 14–15 and small hematomas (≤3 cm) can be treated conservatively. In the case of neurological deterioration, hematoma drainage ± craniectomy should be strongly considered. In comatose patients without brainstem reflexes, formal neurological determination of death should be considered. Comatose patients with preserved brainstem reflexes should be considered for emergency hematoma drainage and suboccipital decompressive craniectomy. Insertion of EVD alone for treatment of cerebellar occupying lesions remains controversial because of the theoretical risk of upward herniation and is not recommended by the AHA guidelines [9]. However, management of recent cohorts of patients with cerebellar infarcts showed that EVD alone is a possible treatment, and can reduce the need for suboccipital decompressive craniectomy [119].

### Intraventricular hemorrhage

IVH occurs in nearly half of ICH patients. Isolated IVH (primary IVH) occurs rarely but more often is the result of secondary extension of a parenchymal hematoma into the ventricular system. The presence of blood in the ventricles can interrupt the normal cerebrospinal fluid (CSF) flow and cause obstructive (noncommunicating) hydrocephalus and increased ICP. Placement of an EVD to drain CSF and monitor ICP should therefore be considered in patients with acute hydrocephalus/IVH and GCS ≤ 8 or with signs of transtentorial herniation [9]. A practical issue arises from the clot burden in the ventricular system and the frequent obstruction of ventricular drain. Techniques such as neuroendoscopy or intraventricular thrombolysis (IVT) have been investigated. The Clot Lysis Evaluation of Accelerated Resolution of Intraventricular Hemorrhage (CLEAR-IVH) trial demonstrated that the use of low-dose recombinant tissue plasminogen activator (r-tPA) had an acceptable safety profile in patients with IVH, as well as being beneficial in accelerating the removal of clot from the ventricular system [120]. The phase III CLEAR-IVH III trial [121], comparing the use of EVD combined with intraventricular injection of r-tPA to EVD plus intraventricular injection of normal saline (placebo) for the treatment of IVH, has been completed (500 subjects enrolled from 73 sites between 2009 and 2014) and preliminary results have been presented recently at the International Stroke Conference (ISC) 2016. The primary outcome of dichotomized mRS 0–3 vs 4–6 at 180 days was not significantly different between the two groups, but the treatment was associated with a 10 % reduction in mortality without increasing the number of patients in a vegetative state or with severe disability. The CLEAR-IVH III researchers also reported that patients with larger clots and more than 20 ml of blood removed showed a significant improvement in functional outcome. In terms of safety, symptomatic bleeding was not more frequent in the alteplase group, and it was associated with a reduction in bacterial ventriculitis (7 % vs 12 %, *p* = 0.05) (official publication of results awaited) [122]. Li et al. [123], in a systematic review and meta-analysis of 11 studies including five RCTs (680 patients), found that the neuroendoscopy + EVD approach seemed to be better than the EVD + IVT approach in terms of mortality, effective hematoma evacuation rate, good functional outcome, and the ventriculoperitoneal shunt dependence rate.

### Minimally invasive surgery

New approaches for hematoma drainage have emerged in the last decade, including stereotactic aspiration of clot ± thrombolysis or endoscopic procedures. Overall, minimally invasive surgery has been associated with improvement in clot removal compared with standard surgical techniques [124, 125]. Cho et al. [126] compared three approaches (neuroendoscopy vs stereotactic aspiration vs craniotomy) in a randomized trial of 90 noncomatose patients with ganglionic hematomas. There was no difference in mortality but patients treated endoscopically had better functional outcomes within 6 months of surgery as assessed by functional independence measure score, Barthel index score, and muscle power. A recent systematic review and meta-analysis showed that death or dependence is significant reduced by minimally invasive surgery when compared with medical management or conventional craniotomy [127]. The Minimally Invasive Surgery Plus rt-PA for ICH Evacuation Phase III (MISTIE III) trial (ClinicalTrials.gov NCT01827046) is currently assessing the usefulness of stereotactic catheter placement into intraparenchymal hematomas followed by direct injection of r-tPA for 3 days and aspiration.

## Quality in acute stroke care

There is evidence for the benefit of stroke units [70] with a significant positive impact on long-term outcome (i.e., decreased morbidity and mortality). Medical complications such as fever, hyperglycemia, VTE, and dysphagia are still not universally optimally managed [128]. Hyperthermia (temperature > 37.5 °C), hyperglycemia, and dysphagia occur in 20–50 %, up to 50 %, and between 37 and 78 % of patients, in the first days after the stroke, respectively.

A. Hyperglycemia is common in patients presenting with ICH and is associated with poor outcomes (hematoma expansion, increased edema, death, or severe disability) [129–132]. The optimal glucose level and the best hyperglycemia management strategy remain to be elucidated. However, both hypoglycemia (<70 mg/dl or < 3.9 mmol/L) and hyperglycemia (>180 mg/dl or 10 mmol/L) should be avoided [9].
B. Temperature. Fever is a common occurrence affecting between 30 and 50 % of patients with ICH, and is independently associated with poor outcomes [133, 134]. The presence of IVH is the main risk factor for fever not explained by infections or drugs [135]. There are no available data from RCTs addressing the role of induced normothermia after ICH. At this point, the suggested goal is to keep a core temperature below 37.5–38 °C [9]. One pilot study (iCOOL1) randomized 20 patients with acute stroke to induced normothermia (cold saline infusion (4 °C, 2 L at 4 L/hour) vs nasopharyngeal cooling (60 L/minute for 1 hour)). A high incidence of serious adverse events (seven in total) was reported, suggesting that safety of cooling in acute stroke patients awaits evaluation in future trials [136].
C. Prevention of VTE. Patients with ICH are considered at high risk of VTE, reported to be up to 4-fold higher than in patients with ischemic stroke [137]. Initial prophylaxis utilizes intermittent pneumatic compression devices to be positioned at the time of hospital admission (Strong recommendation and high-quality evidence) [138, 139], followed by pharmacological prophylaxis with UFH or LMWH, initiated after documented cessation of bleeding by imaging [140]. This is often considered to be 24–48 hours from the initial hemorrhage [139].
D. Dysphagia is common after stroke, and its reported incidence varies from 37 to 78 %, depending on the technique used for detection [141]. Dysphagia is associated with increased risk for pneumonia/pneumonitis (RR 3.17, 95 % CI 2.07-4.87) [141]. Occurrence of aspiration pneumonia can be reduced by formal dysphagia screening (e.g., water swallow test) from 5.4 % to 2.4 % (3 % absolute risk reduction) [142]. According to the AHA/ASA guidelines, “a formal screening procedure for dysphagia should be performed in all patients before the initiation of oral intake to reduce the risk of pneumonia” (Class I; Level of Evidence B) [9].
E. Anemia. Hemoglobin levels on admission and nadir levels seem to be associated with outcome after ICH [143, 144]. However, there is currently no universally accepted ideal hemoglobin level in this population.
F. Seizure prophylaxis. Seizure frequency has been reported between 8.1 and 10.6 % in patients with ICH, with status epilepticus occurring in 1–2 % of patients [9]. Lobar hemorrhages are independent predictor of early and late seizures (i.e., occurring more than 2 weeks after ictus). However, prophylactic use of anticonvulsants in ICH patients is associated with worse outcomes. Phenytoin has been associated with increased side effects and worse outcomes [145]. The current AHA ICH guidelines do not recommend the use of prophylactic anticonvulsants [9]. Continuous EEG has become a monitor that is used in the management of patients with ICH. Claassen et al. [146] reviewed the records of 102 consecutive patients who underwent continuous EEG monitoring after ICH. They found that one-third of ICH patients developed seizures, with more than half having only electroencephalographic evidence of seizures. They also found a correlation between electroencephalographic seizures and expanding hemorrhages, and also between periodic discharges in patients with cortical ICH and poorer outcomes [146]. Patients with ICH presenting with a decreased level of consciousness out of proportion to the clinical expected level of neurologic impairment may benefit from continuous EEG monitoring, which can be used to trigger or modify therapy [9].

Management of increased ICP is a topic beyond the scope of this review, and we refer to very informative articles published recently [147]. In general, ICP management strategies include: head of bed elevation between 30 and 45°, CSF drainage through EVD, analgesia and sedation, normocapneic ventilation, and administration of hypertonic solutions (e.g., hypertonic saline or mannitol). In refractory cases, hypothermia, barbiturates, or decompressive craniectomy can be attempted [147].

## Challenges in prognostication

Several clinical ICH grading scales have been published with the ultimate goal of risk stratification and prognostication [68]. However, a confounder in the accuracy of these scales is the impact of withholding or withdrawal of life support (WOLS). Most ICH patients die in the acute phase because of WOLS [148, 149]; especially in patients with a high ICH score, WOLS is associated with early death [148, 149]. Diringer et al. [150] showed in a large series of more than 2000 mechanically ventilated patients admitted to a neuroscience ICU that older or comatose patients were more likely to have their life support withdrawn. Because of the uncertainty in prognostication in the early phase, early aggressive management is recommended after ICH, and treatment limitations should not be based solely on prognostic models [9].

## Conclusion

Spontaneous ICH is a neurological emergency associated with high mortality and morbidity. Key management issues include prompt etiologic diagnosis, reversal of anticoagulation, consideration of surgical management, and control of blood pressure. The clinical scenario and local practice will influence choice of therapeutic environment. As of March 2016, there were 581 ClinicalTrials.gov registered trials evaluating therapeutic opportunities in ICH. Early prognostication should not be attempted except when clear signs of nonconfounded irreversible brain damage are present, such as an absence of brain stem reflexes.

## Abbreviations

AAICH, anticoagulant-associated intracerebral hemorrhage; APT, antiplatelet therapy; ATACH, Antihypertensive Treatment of Acute Cerebral Hemorrhage; BP, blood pressure; CBF, cerebral blood flow; CMRO_2,_ cerebral metabolic rate of oxygen; CPP, cerebral perfusion pressure; CSF, cerebrospinal fluid; CT, computed tomography; CTA, computed tomography angiography; DSA, digital subtraction angiography; ED, emergency department; EVD, external ventricular drain; FFP, fresh frozen plasma; GCS, Glasgow Coma Scale; ICH, intracerebral hemorrhage; ICP, intracranial pressure; INR, International Normalized Ratio; IVH, intraventricular hemorrhage; IVT, intraventricular thrombolysis; MRI, magnetic resonance imaging; mRS, modified Rankin Scale; NIHSS, National Institutes of Health Stroke Scale; OEF^max,^ maximum oxygen extraction fraction; OR, odds ratio; PCC, prothrombin complex concentrate; rFVIIa, recombinant activated factor VII; r-tPA, recombinant tissue plasminogen activator; SBP, systolic blood pressure; VTE, venous thromboembolism; WOLS, withdrawal of life support

## Additional file

Additional file 1: Table S1. Etiology of spontaneous ICH. **Table S2.** Initial management. **Table S3.** Composition of some four-factor PCCs. **Table S4.** Ongoing studies on ICH management, and **Table S5.** Hematoma expansion scores. (DOCX 28 kb)
