# Supplementary material for: The critical care management of spontaneous intracranial hemorrhage: a contemporary review
Source: Crit Care. 2016 Sep 18;20:272. doi: 10.1186/s13054-016-1432-0 (PMC5027096; doi:10.1186/s13054-016-1432-0)
Supplement: Additional file 1: Table S1. — Etiology of spontaneous ICH. Table S2. Initial management. Table S3. Composition of some four-factor PCCs. Table S4. Ongoing studies on ICH management, and Table S5. Hematoma expansion scores. (DOCX 28 kb) [file 13054_2016_1432_MOESM1_ESM.docx]

**Table S1** – Etiology of Spontaneous Intracerebral Hemorrhage (ICH)

| **Primary** | **Secondary** |
| --- | --- |
| - Hypertension  (most common cause) - Cerebral amyloid angiopathy (most common cause of lobar ICH, especially in the elderly) - Sympathomimetic drugs (e.g cocaine, methamphetamine) - Coagulopathy (bleeding disorders, antithrombotic agents, thrombolytic therapy, anticoagulants) | - Vascular malformations - Arteriovenous malformation - Cavernous malformation - Cerebral aneurysms - Dural arteriovenous fistula - Hemorrhagic transformation - Ischemic stroke - Post-tPA - Dural venous sinus thrombosis (hemorrhagic venous infarction) - Primary intracranial tumours - Glioblastoma multiforme - Oligodendroglioma - Metastases of primary extracranial tumours - Malignant melanoma - Bronchial carcinoma - Thyroid carcinoma - Hypernephroma - Breast - Infections (especially “mycotic” aneurysms – endocarditis-related septic cerebral emboli –, aspergillosis, and herpes simplex encephalitis) - Primary or secondary CNS vasculitis (rare cause) - Moyamoya (rare cause) |

Table S2 – Initial Management

| 1. *ABC.* Initial assessment and stabilization of airway, breathing, and circulation. 2. *Rapid Neuroimaging.* CT or MRI can be used to differentiate ICH from an ischemic stroke. 3. *Neurologic assessment to determine baseline severity*. (i.e. Glasgow Coma Scale (GCS), NIHSS and /or ICH score calculation). 4. *Blood pressure management* (*AHA/ASA guidelines for Blood Pressure Management after ICH* - NEW Guidelines 2015). For ICH patients presenting with SBP between 150 and 220 mmHg and without contraindication to acute BP treatment, acute lowering of SBP to 140 mm Hg is safe and can be effective for improving functional outcome. For ICH patients presenting with SBP > 220 mm Hg, it may be reasonable to consider aggressive reduction of BP to < 140 mmHg with a continuous intravenous infusion and frequent BP monitoring. 5. *Clinical history and laboratory evaluation of coagulopathy*. Evaluate the use of anticoagulants or history of bleeding disorder. Measure platelets count, partial thromboplastin time (PTT), and international normalized ratio (INR). 6. *Aggressive correction of coagulation derangements*. If coagulation abnormalities are detect, they should be quickly and aggressively correct. 7. *Admission to Neurointensive Care Unit (NICU) and frequent neurological assessment.* Ideally, neuro checks every hour in a NICU environment, what would allow for timely detection of early neurological deterioration.   8. *Management of increased intracranial pressure.* |
| --- |

*Table S3 – Factor composition of some of the available commercial preparations of 4PCC*

| *Name* | *Beriplex 500 (Kcentra)* | Octaplex 500 | Cofact 500 | Confidex 500 |
| --- | --- | --- | --- | --- |
| *Manufacturer* | *CSL Behring* | Octapharma | Sanquin | CSL Behring |
| *Factor II* | *380–800* | *280-760 IU* | *280 – 700* | *400 – 960* |
| *Factor IX* | *400–620* | *500 IU* | *500* | *400 – 620* |
| *Factor X* | *500–1020* | *360-600 IU* | *280 – 700* | *440 – 1200* |
| *Factor VII* | *200–500* | *180-480 IU* | *140 – 400* | *200 – 500* |
| *Protein C and S* | *Yes* | *Yes* | *Yes* | *Yes* |
| *Heparin* | *Yes* | *No* | *No* | *No* |

*Table S4 – Evidenced based summary of ICH management*

| *ClinicalTrials.gov*  *(study name)* | *Country* | *Current Status* | *Population* | *Intervention* | *Primary outcome* | *Sample size* |
| --- | --- | --- | --- | --- | --- | --- |
| *Blood pressure control* | | | | | | |
| *NCT02281838*  *(ICH-ADAPT II)* | *Canada* | *Recruiting* |  | *SBP <180 mmHg versus SBP <140 mmHg within one hour after ranzomization* | *Diffusion-weighted imaging (DWI) lesion frequency* | *270* |
| *Temperature Control* | | | | | | |
| *NCT01584167*  *(*iCOOL 2 ) | *Germany* | *Recruiting* | *Stroke (including hemorrhagic)* | *Cold infusions versus EMCOOLS Flex.Pads* | *Brain temperature* | *20* |
| *NCT00751634* | *United States* | *Completed, not published* | *Neurological patients* | *Gaymar Rapr-Round* | *Core Temperature* | *20* |
| *NCT01607151*  *(TTM-ICH)* | *United States* | *Recruiting* | *Intracerebral Hemorrhage* | *Normothermia (36-37°C) versus hypothermia (32-34°C)* | *Severe adverse events* | *50* |
| *NCT02078037*  *SNICH* | *United States* | *Ongoing, not recruiting* | *Intracerebral Hemorrhage* | *Arctic Sun® 5000 (target 35.5 - 37.5^o^C) versus Acetaminophen + cooling blanket (target < 38.5^o^C)* | *MRI measurement of relative perihematomal edema* | *100* |
| *Transfusion/Coagulation* | | | | | | |
| *NCT00928915*  *(INCH)* | *Germany* | *Unknown* | *Intracerebral Haemorrhage* | *Prothrombin complex concentrate versus fresh frozen plasma* | *INR ≤ 1.2 within 3 hours after start of drug infusion* | *74* |
| *NCT00699621* | *Finland* | *Unknown* | *Intracerebral Hemorrhage using Platelet Inhibitors* | *Standard care versus platelets* | *Hematoma growth within 24 h measured as increase in hematoma volume observed by head CT* | *100* |
| *NCT00222625* | *Italy* | *Unknown* | *Intracerebral Hemorrhage using Platelet Inhibitors or anticoagulants* | *Factor VIIa + (vit K in anticoagulated patients) versus plasma or prothrombin complex concentrate* | *Change in ICH volume from prior to dosing to 24 hours* | *32* |
| *NCT01359202*  *(SPOTLIGHT)* | *Canada* | *Recruiting* | *Intracranial hemorrhage with spot sign present* | *Factor VIIa versus placebo* | *ICH size in 24h* | *110* |
| *Surgical Management* | | | | | | |
| *NCT02135783*  *(CARICH)* | *China* | *Recruiting* | *Intracranial hemorrhage in the first 72h, volume 30-100 mL, lobar or basal ganglia location* | *Decompressive versus non-decompressive craniectomy* | *Mortality and disability* | *200* |
| *NCT01827046*  *(MISTIE III)* | *United States* | *Recruiting* | *Intracranial hemorrhage, supratentorial volume ≥ 30 mL* | *Surgical drainage with local rt-PA infusion versus Clinical Management* | *Modified Rankin Scale score at 180 days* | *500* |
| *NCT02258919*  *(SWITCH)* | *Germany* | *Recruiting* | *Intracranial hemorrhage, volume of ≥30 ml and ≤100 ml* | *Decompressive craniectomy versus clinical management* | *Modified Rankin Scale score at 180 days* | *300* |

*Table S5 – Hematoma expansion scores -* CTA indicates computed tomography angiography; GCS, Glasgow Coma Scale; ICH, intracerebral hemorrhage; INR, international normalized ratio; IVH, intraventricular hemorrhage; NIHSS, National Institutes of Health Stroke Scale; and PREDICT, Predicting Hematoma Growth and Outcome in Intracerebral Hemorrhage Using Contrast Bolus Computed Tomography.

| 9-Point Score | | 24-Point Score (Brain) | | PREDICT A Score | | Predict B Score | |
| --- | --- | --- | --- | --- | --- | --- | --- |
| Component | Points | Component | Points | Component | Points | Component | Points |
| Baseline ICH volume, ml  < 30  30-60  > 60 | 0  1  2 | Baseline ICH volume, ml  ≤ 10  10-20  > 20 | 0  5  7 | GCS  14-15  ≤ 13 | 0  4 | NIHSS  0-4  5-14  ≥ 15 | 0  4  7 |
| Hours from onset to CT  ≤ 6  > 6 | 2  0 | Hours from onset to CT  ≤ 1  > 1-2  > 2-3  > 3-4  > 4-5  > 5 | 5  4  3  2  1  0 | Hours from onset to CT  ≤ 1  > 1-2  > 2-3  > 3-4  > 4-5  > 5 | 5  4  3  2  1  0 | Hours from onset to CT  ≤ 1  > 1-2  > 2-3  > 3-4  > 4-5  > 5 | 5  4  3  2  1  0 |
| Warfarin use  Yes  No | 2  0 | Warfarin use  Yes  No | 6  0 | Warfarin use or INR > 1.5  Yes  No | 6  0 | Warfarin use or INR > 1.5  Yes  No | 7  0 |
| CTA spot sign  Present  Absent  Unavailable | 3  0  1 |  |  | CTA spot sign number  0 spots  1 spot  ≥ 2 spots | 0  4  8 | CTA spot sign number  0 spots  1 spot  ≥ 2 spots | 0  4  9 |
|  |  | IVH extension  Yes  No  Recurrent ICH  Yes  No | 2  0  4  0 |  |  |  |  |
| Total | 0-9 |  | 0-24 |  | 0-23 | 0-28 |  |
